# Structural characterization of PAMK and its protective effect against thymic necroptosis in goslings

**DOI:** 10.1080/01652176.2026.2695104

**Published:** 2026-09-01

**Authors:** Wanyan Li, Baili Lu, Xiangying Zhou, Shirou Pan, Junhao Wei, Bingxin Li, Nan Cao, Yunmao Huang, Wenjun Liu, Xinliang Fu, Yunbo Tian, Danning Xu

**Affiliations:** a College of Animal Science & Technology, Zhongkai University of Agriculture and Engineering, Guangzhou, China; b Guangdong Engineering Technology Research Center of Biosafety and Intelligent Control for Aquatic Animals Diseases, Guangzhou, China; c Faculty of Applied Sciences, Macao Polytechnic University, Macao, China

**Keywords:** PAMK, necroptosis, CTX, thymus, novel-miR-2

## Abstract

PAMK are heterogeneous glycans with immunomodulatory potential, but their structural basis and mechanisms in regulating thymic injury remain unclear. Structural characterization revealed that PAMK is mainly composed of arabinose, galactose, rhamnose, galacturonic acid, and glucose, featuring a dominant backbone consisting of →4)-*α*-D-GalpA-(1→, →4)-*β*-D-Galp-(1→, →2)-*α*-L-Rhap-(1→, and →3,4)-*α*-D-GalpA-(1→ with highly branched side chains. This complex structure provides the biochemical foundation for its bioactivity. To explore its immunoprotective effects, we established a CTX-induced thymic necroptosis model in goslings. CTX markedly disrupted cytokine profiles and activated necroptosis signaling, accompanied by downregulation of novel-miR-2 and upregulation of TRADD and MLKL. Dietary PAMK supplementation significantly restored cytokine homeostasis and inhibited necroptosis pathway activation. Mechanistically, PAMK upregulated novel-miR-2, which negatively regulated TRADD and MLKL expression, thereby attenuating necroptosis signaling. Overexpression of novel-miR-2 suppressed TRADD/MLKL-mediated necroptosis, whereas its inhibition produced the opposite effect. Furthermore, PAMK mitigated oxidative stress and necroptotic cell death induced by TRADD and MLKL overexpression. Collectively, our findings demonstrate that the defined structural features of PAMK underpin its ability to protect against CTX-induced thymic necroptosis by upregulating novel-miR-2 and suppressing the TRADD/MLKL axis. This work links PAMK's composition to its immunoprotective function and highlights its potential as a safe immunoregulatory agent in poultry.

## Introduction

The thymus is one of the most critical central lymphoid organs in the immune system, primarily responsible for the generation and export of mature T lymphocytes. These cells play an irreplaceable role in maintaining immune homeostasis and establishing immune tolerance (Ashby and Hogquist 2024; Baldwin and Robey 2024). In poultry, particularly during the early developmental stage of goslings, the thymus is not yet fully mature in either structure or function, rendering it highly susceptible to environmental stress, pathogen infection, and immunosuppressive agents. Such damage often results in impaired T-cell differentiation, thymic atrophy, and immune dysfunction, ultimately reducing disease resistance and production performance (Francelin et al. 2021; Ruiz Perez et al. 2024). Therefore, investigating the mechanisms and protective strategies of necroptosis in gosling thymus is of great significance for ensuring the healthy breeding of goslings and improving the overall immune level of the flock.

The thymus is highly sensitive to pathological insults, and one of its key manifestations of injury is the activation of necroptosis. Necroptosis is a programmed form of necrosis driven by the RIPK1-RIPK3-MLKL axis. Unlike apoptosis, it disrupts plasma membrane integrity, induces organelle swelling and cytoplasmic vacuolization, and causes the release of intracellular contents, thereby exacerbating inflammation and tissue damage (Pasparakis and Vandenabeele 2015). Recent studies have demonstrated that thymocytes undergo necroptotic activation under various pathological conditions, which severely disrupts thymic architecture and T-cell maturation (Jain et al. 2021; Su et al. 2024). Therefore, thymic necroptosis itself—rather than general ‘thymic injury’—represents a pivotal pathological feature and a meaningful mechanistic target for intervention. In this study, the immunosuppressant CTX was employed to induce immune stress and establish a reproducible experimental model of thymic pathology (Dionisio et al. 2022). Importantly, our focus is not on CTX-induced toxicity per se, but rather on necroptosis as a fundamental pathological process occurring in the thymus under stress conditions.

Increasing evidence further suggests that non-coding RNAs, particularly microRNAs (miRNAs), are key regulators of necroptosis and immune signaling. In avian immunology, miRNAs have been implicated in T-cell development, cytokine secretion, and tolerance (Taheri et al. 2020; Dosil et al. 2022). By binding to target mRNAs, miRNAs regulate their stability and translational efficiency, thereby modulating diverse physiological and pathological processes (Diener et al. 2022). Several miRNAs, such as miR-21, miR-34a, and miR-155, directly target components of the necroptosis pathway, thereby influencing both cell death and inflammatory responses (Shirjang et al. 2019). Nevertheless, whether necroptosis contributes to thymic injury in goslings and whether PAMK can suppress necroptosis in thymocytes remain unclear. Moreover, it has not yet been determined whether novel-miR-2 directly targets genes in the necroptosis pathway. Addressing these questions would provide valuable insights into the molecular mechanisms of immune injury and reveal potential therapeutic targets.

Polysaccharide of *Atractylodes macrocephala* Koidz (PAMK) have attracted substantial attention for their immunomodulatory and antioxidative activities. Structural features such as monosaccharide composition and glycosidic linkages are critical determinants of their recognition by immune receptors and thus of their biological effects (Zhu et al. 2018). Extensive studies have documented the diverse biological functions of PAMK, including immunoenhancement, antioxidation, anti-inflammation, intestinal barrier protection, and stress resistance (Liu et al. 2022). Notably, our previous work has shown that PAMK attenuates CTX-induced thymic injury in goslings by alleviating lipid peroxidation and ferroptosis (Zhou et al. 2022). However, while ferroptosis highlights iron-dependent metabolic damage, it is well established that multiple programmed cell death pathways concurrently drive tissue injury. Specifically, necroptosis—a lytic cell death pathway characterized by the RIPK1/RIPK3/MLKL axis—is the primary driver of membrane rupture and the subsequent massive release of pro-inflammatory contents (DAMPs), triggering a vicious cycle of ‘necroinflammation’. Whether PAMK can halt this necroptosis-driven inflammatory cascade remains completely unknown. Furthermore, our previous investigations lacked a precise structural characterization of the polysaccharide. Despite various reports on PAMK's immunomodulatory effects (Xu et al. 2014; Li et al. 2025), the exact chemical structure of our active fraction and how this specific structure regulates novel epigenetic targets to influence thymic necroptosis have not been elucidated. Therefore, exploring the structural basis of PAMK and its specific regulation of the necroptotic machinery represents a critical and highly novel scientific gap.

Based on this rationale, the present study first characterized the structural features of PAMK and then investigated its effects on thymic necroptosis in a gosling model of immune stress. Specifically, we evaluated necroptosis-associated signaling, miRNA involvement, and the protective mechanisms of PAMK. Our findings provide novel insights into necroptosis as a central thymic pathology and establish PAMK as a promising immunoprotective candidate targeting necroptosis in poultry.

## Materials and methods

### Reagents

PAMK (purity ≥ 95%, catalog number: CY201216) was purchased from Yangling Ciyuan Biotechnology Co., Ltd. (Yangling, China). PowerUp™ SYBR™ Green Master Mix (catalog number: A25742) was obtained from Thermo Fisher Scientific (USA). Reverse transcription kit (catalog number: RR036A) was purchased from Takara Bio Inc. (Japan). Trizol RNA isolation reagent (catalog number: 15596-026) was obtained from Applied Biosystems (USA). riboSCRIPT Reverse Transcription kit (catalog number: C11027-3) was purchased from RiboBio Co., Ltd. (Guangzhou, China). Anti-GAPDH Mouse mAb (catalog number: 60004-1-1g) and HRP-conjugated Affinipure Goat Anti-Mouse IgG(H+L) (catalog number: SA00001-1) were obtained from Proteintech (Wuhan, China). Anti-MLKL Rabbit mAb (catalog number: A19685), Anti-Phospho-MLKL Rabbit pAb (catalog number: AP0949), Anti-RIPK3 Rabbit pAb (catalog number: A5431), Anti-Phospho-RIP3 Rabbit pAb (catalog number: AP1260), and Anti-RIPK1/RIP Rabbit pAb (catalog number: A7414) were obtained from Abclonal (Wuhan, China). Anti-TRADD Rabbit pAb (catalog number: bs-1202R), Anti-FADD Rabbit pAb (catalog number: bs-0511R), and Anti-CYLD Rabbit pAb (catalog number: bs-2756R) were purchased from Bioss Biotechnology Co., Ltd. (Beijing, China). Anti-Caspase-8 Rabbit pAb (catalog number: WL00659), Anti-TNFR1 Rabbit pAb (catalog number: WL01414), Anti-TGFβ Rabbit pAb (catalog number: WL02998), Anti-mature IL-1β Rabbit pAb (catalog number: WL00891), Anti-mature IL-6 Rabbit pAb (catalog number: WL02841), and Anti-mature IL-10 Rabbit pAb (catalog number: WL03088) were purchased from Wanleibio (Shenyang, China). Anti-TNFα Rabbit pAb (catalog number: WL01581), Anti-mature IL-2 Rabbit pAb (catalog number: PAA073Ga01), Anti-mature IL-4 Rabbit pAb (catalog number: PAA077Ga01), and Anti-IFNγ Rabbit pAb (catalog number: PAA049Ga01) were obtained from Cloud-Clone Corp. (Wuhan, China). HRP-conjugated Affinipure Goat Anti-Rabbit IgG(H+L) (catalog number: SA00001-2) was also obtained from Proteintech (Wuhan, China). ELISA kits for IL-1β (catalog number: ZK-C6332) and IL-6 (catalog number: ZK-G7318) were obtained from ZIKER (Shenzhen, China). ELISA kits for IL-2 (catalog number: XY-ELA05691), IL-4 (catalog number: XY-ELA05680), IL-10 (catalog number: XY-ELA05678), IFN-*γ* (catalog number: XY-ELA0576), and TGF-*β* (catalog number: XY-ELA05696) were purchased from Xinyu Biotech (Shanghai, China). Reactive Oxygen Species Assay Kit (catalog number: S0033S), Glutathione Peroxidase Assay Kit (catalog number: S0056), and Total Superoxide Dismutase Assay Kit (catalog number: S0101S) were purchased from Beyotime Biotechnology (Shanghai, China). Malondialdehyde (MDA) Assay Kits (catalog numbers: A003-4-1 and A003-1) were obtained from Nanjing Jiancheng Bioengineering Institute (Nanjing, China). RPMI-1640 medium (catalog number: C22400500BT), Fetal Bovine Serum (FBS; catalog number: 10270106), and Opti-MEM Reduced Serum Medium (catalog number: 31985079) were obtained from Gibco (USA). Lipofectamine™ 3000 Transfection Reagent (catalog number: L3000015) was purchased from Thermo Fisher Scientific (USA). NADPH Regeneration System and Chicken Liver Microsomes (catalog number: C10001) were obtained from Puli Technology Co., Ltd. (Wuhan, China).

### Polysaccharide extraction and purification

Raw materials were defatted with ethanol and extracted with hot water. The combined extracts were ethanol-precipitated, enzymatically treated to remove impurities, and further purified by ion-exchange and gel-filtration chromatography. The major carbohydrate fractions were collected, desalted, lyophilized, and their purity was assessed by the phenol–sulfuric acid assay.

### Monosaccharide composition analysis

Monosaccharide composition was analyzed by ion chromatography. Samples were hydrolyzed with 2 M trifluoroacetic acid (121 °C, 2 h), dried under nitrogen, washed with methanol, and redissolved in ultrapure water. Standard monosaccharides (Sigma-Aldrich) were used to generate calibration curves. Chromatographic separation was performed on a Thermo ICS-5000+ system with a CarboPac™ PA20 column and electrochemical detection under a NaOH/NaOAc gradient. Monosaccharides were identified by comparison with standards and quantified using external calibration (R^2^ ≥ 0.99).

### Polysaccharide linkage analysis (methylation-GC-MS)

Polysaccharide linkage analysis was performed by carboxyl reduction and permethylation followed by GC–MS. Briefly, ~5 mg sample was dissolved in 1 mL water, activated with 1 mL 100 mg/mL CMC for 2 h, and reduced with NaBH₄ or NaBD₄ (30 mg/mL, 3 h). After dialysis and lyophilization, the sample was dissolved in 500 μL DMSO, treated with 1 mg NaOH (30 min), and methylated with 50 μL CH₃I (1 h). The permethylated product was hydrolyzed with 2 M TFA (121 °C, 90 min), reduced with 1 M NaBD₄ in 2 M NH₄OH (2.5 h), and acetylated with 250 μL acetic anhydride (100 °C, 2.5 h). The resulting partially methylated alditol acetates were analyzed by GC–MS (Agilent 7890A–5977B, BPX70 column, 30 m × 0.25 mm × 0.25 μm; injection 1 μL, split 10:1; temperature program: 140 °C for 2 min, then 3 °C/min to 230 °C, hold 3 min; EI-MS, m/z 50–350). Glycosidic linkages were identified by characteristic fragmentation patterns and quantified by relative molar ratios.

### NMR spectroscopy

Polysaccharides were dissolved in D₂O at ≥40 mg/mL and transferred to NMR tubes (0.5 mL). Spectra were acquired on a Bruker AVANCE NEO 500 MHz spectrometer at 25 °C using a QXI 5 mm probe. One-dimensional (^1^H, ^13^C) and two-dimensional (COSY, HSQC, HMBC, NOESY) spectra were collected to resolve glycosidic linkages and configurations. Data interpretation was based on correlation of proton and carbon signals across the six standard spectra.

### Animals and treatment

A total of 40 one-day-old Magang goslings (with an equal number of males and females) were purchased from Qingyuan Jinyufeng Goose Industry Co., Ltd. and randomly assigned to four groups (*n* = 10 per group): control (CON), PAMK, CTX, and PAMK+CTX. After a 3-day adaptation period, the CON and CTX groups were fed a basal diet, while the PAMK and PAMK+CTX groups received a diet supplemented with 400 mg/kg PAMK. On days 19–21, goslings in the CTX and PAMK+CTX groups were intramuscularly injected with CTX at 40 mg/kg body weight (BW) daily, whereas the CON and PAMK groups received sterile saline. The CTX-induced immunosuppression model was established as previously described (Li et al. 2018). On day 28, all goslings were euthanized with sodium pentobarbital (100 mg/kg BW, i.p.), and blood and thymus samples were collected for analysis. All experimental procedures were approved by the Ethics Committee of Zhongkai University of Agriculture and Engineering (ZK202204-20).

### Isolation and culture of thymic lymphocytes

Thymuses were aseptically collected from 15-day-old Magang goslings, surface-sterilized with 0.2% benzalkonium bromide, and processed under sterile conditions. Tissues were dissociated through a 70 μm strainer to obtain single-cell suspensions, which were layered onto lymphocyte separation medium and centrifuged to isolate thymocytes. After red blood cell lysis and washing, cells were resuspended in RPMI 1640 supplemented with 10% FBS and 1% penicillin–streptomycin, adjusted to 3 × 10^6^ cells/mL, and cultured at 39 °C with 5% CO₂. For the PAMK group, cells were pretreated with 25 μg/mL PAMK for 6  h before subsequent assays.

### Bioactivation of CTX and thymocyte treatment

Because CTX requires metabolic activation, it was incubated with chicken liver microsomes in the presence of an NADPH-regenerating system. Briefly, CTX (1 mM) was prepared in a 1 mL mixture containing RPMI 1640, Solution A, Solution B, and liver microsomes (final protein concentration 0.5 mg/mL), preheated at 37 °C and incubated for 30 min. After sequential centrifugation (3,000 rpm, 10 min; 10,000 rpm, 5  min), the supernatant containing metabolized CTX was collected. Primary goose thymocytes were cultured at 39 °C with 5% CO₂ and divided into four groups: CON, PAMK, CTX, and CTX + PAMK. Cells in the CTX group were treated with metabolized CTX at a final concentration of 50 μM, while the PAMK groups were pretreated with PAMK as indicated (Table S1).

### Establishment of novel-miR-2 overexpression and inhibition models in thymocytes

Primary goose thymocytes were cultured at 39 °C with 5% CO₂ for 6 h prior to transfection to establish novel-miR-2 overexpression and inhibition models. Novel-miR-2 mimics, inhibitors, and their corresponding negative controls were designed and synthesized by RiboBio Co., Ltd., and the sequences or catalog numbers are provided in Table S2. For each transfection (performed in triplicate), miRNA products were first diluted in RNase-free water to a stock concentration of 20 μM. Mimic or inhibitor working solutions were prepared by diluting 5 μL of each in 125 μL Opti-MEM, and Lipofectamine 3000 (5 μL) was similarly diluted in 125 μL Opti-MEM. The two solutions were combined and incubated for 15 min at room temperature to allow complex formation before being added dropwise to the cells. Plates were gently rocked to ensure even distribution, and cells were incubated for 24 h at 39 °C in a 5% CO₂ incubator prior to subsequent analyzes.

### Establishment of thymocyte models overexpressing MLKL and TRADD

Primary thymocytes were isolated from goslings and cultured in a cell incubator at 39 °C with 5% CO₂ for 6 h before transfection. Transfection was performed using Lipofectamine™ 3000 (Thermo Fisher Scientific) according to the manufacturer's instructions. For each transfection, 5 μL Lipofectamine 3000 was diluted in 125 μL Opti-MEM (serum-free). In parallel, 5 μL P3000 and DNA (1 μg/mL) were diluted in 125 μL Opti-MEM. The two solutions were combined, gently mixed, and incubated for 15 min at room temperature, then added to cells. After 12 h, medium was replaced with fresh complete medium and cells were cultured for an additional 24 h to allow CCL5 secretion. Supernatants were cleared (1,500 rpm, 5 min) and applied to a second batch of cells. After 24 h to allow CCL5-GAG interaction, cells were harvested for RNA and protein extraction. The specific groupings are shown in Table S3.

### Transmission electron microscopy

For ultrastructural analysis, cells were fixed in 2.5% glutaraldehyde in phosphate buffer at 4 °C, post-fixed with 1% osmium tetroxide, and contrasted with 4.8% uranyl acetate. Samples were dehydrated, embedded, and ultrathin sections (50–70 nm) were prepared. Sections were mounted, washed, and post-stained with lead citrate before observation. Ultrastructural changes were examined using TEM.

### Detection of oxidative stress-related indicators

The levels of malondialdehyde (MDA), superoxide dismutase (SOD), and glutathione peroxidase (GSH-px) in thymocyte were measured using the oxidative stress indicator assay kits from Beyotime Biotechnology, following the manufacturer's instructions. The content of reactive oxygen species (ROS) was determined using the ROS detection kit from Beyotime Biotechnology, and the results were analyzed using a flow cytometer.

### Quantitative real-time PCR

Total RNA was extracted from thymus (50 mg) or cultured thymocytes using TRIzol (Invitrogen) according to the manufacturer's instructions. Tissues were homogenized with a bead mill, while cells were washed in PBS and lysed directly. RNA was isolated by chloroform–isopropanol extraction, washed with 75% ethanol, and dissolved in RNase-free water. Quantity and purity were assessed by micro-spectrophotometry. For mRNA analysis, 500 ng RNA was reverse-transcribed with the PrimeScript RT Master Mix kit (Takara). For miRNA, 1 μg RNA was reverse-transcribed using the riboSCRIPT kit with stem-loop primers specific for U6 and novel-miR-2. qPCR was performed with gene-specific primers for goose cytokines and necroptosis-related genes (designed from NCBI sequences) and novel-miR-2 primers reported previously (Li et al. 2021). All primers were synthesized by BGI and are listed in Table S4.

### Western blot

Total protein from goose thymus or cultured thymocytes was extracted using RIPA lysis buffer supplemented with protease and phosphatase inhibitors. Equal amounts of protein (20 μg per lane) were separated on SDS-PAGE gels and transferred to methanol-activated PVDF membranes. Membranes were blocked with 5% non-fat milk in PBST for 3 h at room temperature and incubated overnight at 4 °C with primary antibodies. After three washes, membranes were incubated with HRP-conjugated secondary antibodies for 1 h at 37 °C. Protein bands were visualized using an ECL substrate and imaged with a chemiluminescence detection system. Band intensities were quantified using ImageJ software.

### Bioinformatic prediction and analysis of novel-miR-2 target genes

Total RNA extracted from goose thymus was used to construct high-quality sequencing libraries, which were sequenced and processed to obtain clean reads. Unannotated reads were aligned to the reference genome using Bowtie, and candidate miRNAs were identified with miRDeep2. Potential target genes of novel-miR-2 were predicted using TargetFinder. Functional annotation of predicted targets was performed by comparing sequences against the NR (https://ftp.ncbi.nlm.nih.gov/blast/db/FASTA/), SwissProt (http://us.expasy.org/sprot/), GO (http://www.geneontology.org/), KEGG (https://www.kegg.jp/), and COG/KOG (https://www.ncbi.nlm.nih.gov/COG/, http://www.ncbi.nlm.nih.gov/KOG/) databases. The binding sites between novel-miR-2 and its predicted targets were further analyzed using BiBiServ2 (RNAhybrid, https://bibiserv.cebitec.uni-bielefeld.de/rnahybrid).

### Statistical analysis

Experimental data were analyzed using IBM SPSS Statistics 26.0. Data from the novel-miR-2 overexpression and inhibition experiments were analyzed using Student's t-test, while other data were evaluated by one-way analysis of variance (ANOVA) followed by Tukey's multiple comparison test. Results are presented as mean ± standard error of the mean (SEM), and *P* < 0.05 was considered statistically significant. Graphs were generated using GraphPad Prism 10.0 and Adobe Illustrator 2020.

## Results

### Purification of PAMK and monosaccharide composition

**Figure 1.**
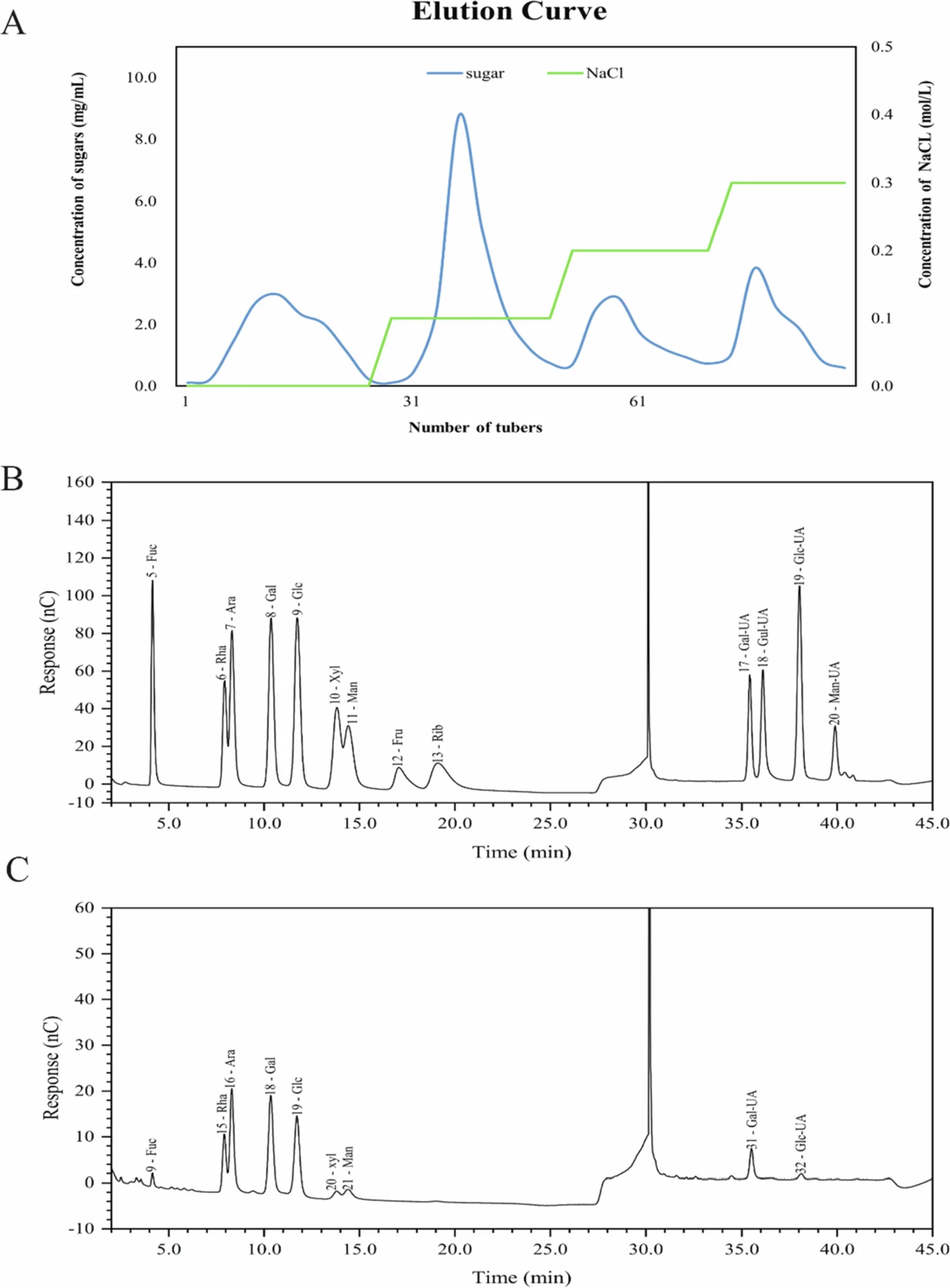
Purification of PAMK and monosaccharide composition. (A) Elution profile of PAMK; (B) Ion chromatogram of standards; (C) Ion chromatogram of monosaccharide composition of PAMK.

The elution profile of PAMK was obtained by ion-exchange chromatography, with total sugar content (blue) and NaCl concentration (green) plotted against the fraction number (Figure 1A). Three major elution peaks were detected: Peak 1 (fractions 7–19), Peak 2 (fractions 34–49), and Peak 3 (fractions 55–67). Peak 2 was further purified by gel filtration chromatography. As shown in Figure 1A, the elution profile—monitored via the phenol-sulfuric acid method for total sugar content—yielded a single, symmetrical peak, confirming the relative purity of the fraction and the absence of significant macromolecular contamination. The monosaccharide composition of PAMK was analyzed using ion chromatography with electrochemical detection and the total sugar content of the purified PAMK fraction was determined to be 63.2% by the phenol-sulfuric acid method (Figure 1B and C, Table 1). PAMK was mainly composed of arabinose (Ara), galactose (Gal), and rhamnose (Rha). Furthermore, the molecular weight distribution was analyzed (Figure S1). The weight-average molecular weight (Mw) was 126.4 kDa and the number-average molecular weight (Mn) was 63.5 kDa, giving a polydispersity index (Mw/Mn) of 1.99. While a PDI below 1.5 defines strict monodispersity, a value of 1.99 is highly characteristic of natural, enzymatically synthesized plant heteropolysaccharides. It reflects a continuous natural variation in chain lengths rather than a mixture of distinct polysaccharide families. These results suggest that PAMK is an acidic heteropolysaccharide with a moderate and acceptable molecular weight distribution, and this composition provided the structural basis for subsequent NMR characterization.

**Table 1.** Monosaccharide composition of PAMK.

Monosaccharide	Abbreviation	%	Molar ratio %
Fucose	Fuc	1.83	1.91
Rhamnose	Rha	17.03	17.76
Arabinose	Ara	20.73	23.65
Galactose	Gal	18.94	18.00
Glucose	Glc	15.01	14.27
Xylose	Xyl	2.83	3.23
Mannose	Man	5.89	5.54
Galacturonic acid	Gal-UA	16.19	14.28
Glucuronic acid	Glc-UA	1.55	1.37
Total sugar content		63.2	

### Linkage composition of PAMK

**Figure 2.**
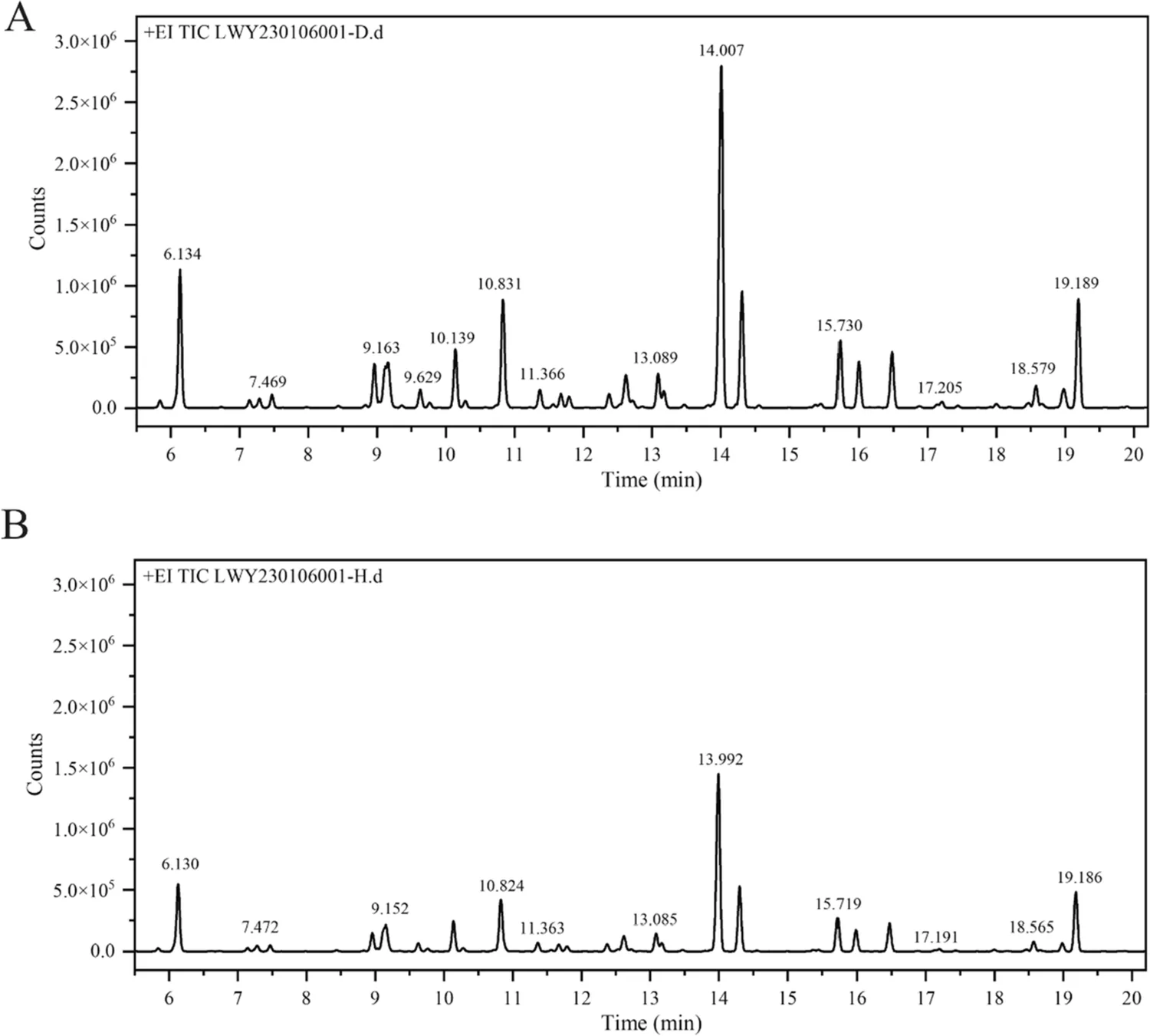
Total ion chromatograms (TIC) of PAMK derivatives. (A) Sample total ion chromatogram (D generation), (B) Sample total ion chromatogram (H generation). +EI: Electron impact ionization source.

In carbohydrate analysis, acidic sugars contain carboxyl groups, and their labile hydrogens readily undergo deuterium exchange in D₂O; under appropriately acidic conditions, this differential exchange can be more clearly manifested in NMR spectra, thereby distinguishing them from neutral sugars. This approach facilitates more precise characterization of complex polysaccharide components, particularly uronic acid residues. As shown in Figure 2, comparison of the two total ion chromatograms (TIC) revealed a strong peak at 14.007 min (Figure 2A), identified as 1,4,5-tri-O-acetyl-2,3,6-tri-O-methyl galactitol, corresponding to the sugar residue 4-Gal(*p*)-UA. Another prominent peak appeared at 6.134 min, identified as 1,4-di-O-acetyl-2,3,5-tri-O-methyl arabinitol, corresponding to the residue t-Ara(f). To provide robust structural evidence, the supporting mass spectra for each major PAMK peak, displaying their characteristic mass fragments (m/z), have been compiled and are presented in Supplementary Figure S3. Additional sugar residues are summarized in Table 2 and Figure S2. Importantly, the completeness of the permethylation reaction—a critical prerequisite for accurate linkage assignment—was verified by the stoichiometric balance of the residues. According to structural carbohydrate principles, the total relative molar ratio of terminal residues (t-Araf and t-Xylp, ~13.42%) was found to be generally consistent with the total molar ratio of branching residues (3,4-Rhap, 4,6-Glcp, and 3,6-Galp, ~10.30%). This internal mathematical agreement indicates that the methylation was essentially complete. Collectively, these data indicate that PAMK is primarily composed of 4-Gal(*p*)-UA and t-Ara(f), and that the presence of uronic acid groups confers an acidic property to its structure.

**Table 2.** The results of methylation analysis of PAMK.

Linkage pattern	Methylated sugar	RT	Mass fragments (m/z)	Molar ratio (%)
t-Ara(f)	1,4-di-O-acetyl-2,3,5-tri-O-methyl arabinitol	6.134	71,87,102,118,129,145,161	12.24
t-Xyl(*p*)	1,5-di-O-acetyl-2,3,4-tri-O-methyl xylitol	7.469	88,101,102,118,119,161,162	1.18
3-Ara(f)	1,3,4-tri-O-acetyl-2,5-di-O-methyl arabinitol	9.629	87,99,113,118,129,201,233	1.19
5-Ara(f)	1,4,5-tri-O-acetyl-2,3-di-O-methyl arabinitol	10.831	87,102,118,129,162,189	10.64
3,4-Rha(*p*)	1,3,4,5-tetra-O-acetyl-6-deoxy-2-O-methyl rhamnitol	11.366	87,99,113,118,129,149,173,275	1.44
3-Gal(*p*)	1,3,5-tri-O-acetyl-2,4,6-tri-O-methyl galactitol	13.089	87,101,118,129,161,202,234	2.60
4-Gal(*p*)-UA	1,4,5-tri-O-acetyl-2,3,6-tri-O-methyl galactitol	14.007	87,99,102,115,118,131,235	17.98
6-Gal(*p*)	1,5,6-tri-O-acetyl-2,3,4-tri-O-methyl galactitol	15.73	87,99,102,118,129,162,189,233	4.91
4,6-Glc(*p*)	1,4,5,6-tetra-O-acetyl-2,3-di-O-methyl glucitol	18.579	85,102,118,127,159,162,201,261	1.58
3,6-Gal(*p*)	1,3,5,6-tetra-O-acetyl-2,4-di-O-methyl galactitol	19.189	87,101,118,129,160,189,234	7.28

### NMR spectrum analysis of PAMK

**Figure 3.**
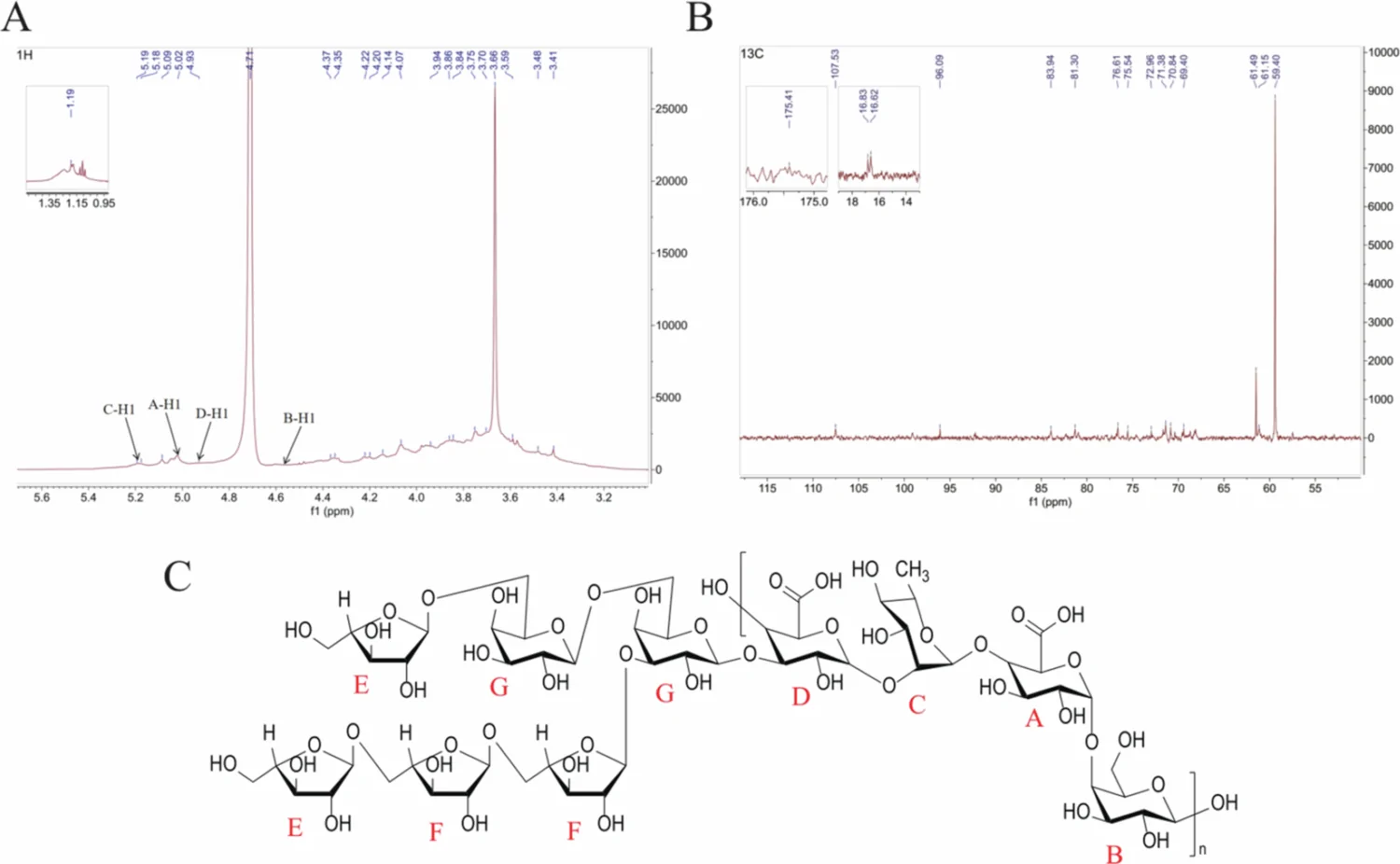
Spectrum analysis of PAMK. (A) ^1^H-NMR spectrum; (B) ^13^C NMR spectrum; (C) The backbone structural diagram of the polysaccharide.

To further clarify the primary structural composition and glycosidic linkages of the major repeating units in PAMK, one- and two-dimensional NMR spectra (1H, 13C and HSQC) were thoroughly analyzed. Multiple distinct anomeric proton and carbon signals were observed, indicating a complex heteropolysaccharide structure composed of various sugar residues. Through comprehensive spatial correlation analysis, a predominant structural backbone (Fragment 1) was successfully identified. The chemical shifts of the major residues (designated A-H) constituting Fragment 1 are detailed in Table 3, while the complete NMR assignments for the remaining independent or minor structural units are provided in Supplementary Table S5. The proposed structure of Fragment 1 is shown in Figure 3C.

**Table 3.** Chemical shifts of ^1^H and ^13^C for the sugar residues.

Code	Glycosyl residues	Chemical shifts(ppm)					
		H1/C1	H2/C2	H3/C3	H4/C4	H5/C5	H6/C6
A	→4)-*α*-D-Gal*p*A-(1→	5.02	3.69	3.93	4.35	4.71	/
		99.07	68.1	68.68	77.79	71.38	175.41
B	→4)-*β*-D-Gal*p*-(1→	4.57	3.61	3.84	4.05	3.64	3.63
		104.2	72.96	73.8	76.54	74.6	61.49
C	→2)-*α*-L-Rha*p*-(1→	5.19	4.06	3.83	3.36	3.73	1.19
		98.59	76.78	69.4	69.59	68.05	16.62
D	→3,4)-*α*-D-Gal*p*A-(1→	4.93	3.85	4.07	4.22	n.d	/
		97.62	69.4	76.77	79.15	n.d	n.d
E	*α*-L-Ara*f*-(1→	5.18	4.15	3.96	4.05	3.66	/
		109.28	81.74	76.61	83.94	59.4	/
F	→5)-*α*-L-Ara*f*-(1→	5.02	4.07	3.88	3.97	3.89,3.74	/
		107.53	81.3	76.77	84.07	66.37	/
G	→6)-*β*-D-Gal*p*-(1→	4.41	3.29	3.5	3.65	3.77	3.93
		103.54	72.86	74.76	68.21	71.38	69.55

For Residue A, a prominent anomeric proton signal at δH 5.02 ppm was clearly observed, which directly correlated to an anomeric carbon signal at δC 99.07 ppm. The proton signals at δH 3.69, 3.93, 4.35, and 4.71 ppm were perfectly correlated to the carbon signals at δC 68.10, 68.68, 77.79, and 71.38 ppm, corresponding to C2 through C5, respectively. Notably, the distinct downfield carbon signal at δC 175.41 ppm confirmed the presence of a carboxyl group at C6. All of these observations proved the structure of residue A, which is assignable to →4)-*α*-D-GalpA-(1→.

For Residue B, an anomeric proton signal emerged at δH 4.57 ppm, showing a clear correlation with the carbon signal at δC 104.20 ppm. The sequential C-H cross-peaks for positions 2 through 6 were explicitly evident: proton signals at δH 3.61, 3.84, 4.05, 3.64, and 3.63 ppm were observed to correlate with carbon signals at δC 72.96, 73.80, 76.54, 74.60, and 61.49 ppm, respectively. This specific profile is characteristic of the →4)-*β*-D-Galp-(1→ linkage.

For Residue C, a characteristic anomeric proton signal was located at δH 5.19 ppm, correlating to δC 98.59 ppm. A prominently upfield proton signal at δH 1.19 ppm was uniquely correlated to a methyl carbon at δC 16.62 ppm, representing the definitive CH3 (C6) group of rhamnose. The signals for C2-C5 (δC 76.78, 69.40, 69.59, and 68.05 ppm) aligned directly with their respective protons at δH 4.06, 3.83, 3.36, and 3.73 ppm, drawing the conclusion that Residue C is →2)-*α*-L-Rhap-(1→.

For Residue D, the anomeric proton and carbon signals were identified at δH 4.93 ppm and δC 97.62 ppm. The signals for C2-C4 (δC 69.40, 76.77, and 79.15 ppm) directly corresponded to their respective protons at δH 3.85, 4.07, and 4.22 ppm, indicating its structure as the branching point →3,4)-*α*-D-GalpA-(1→.

The proposed structure was further supported by COSY, NOESY, and HMBC spectra. Based on the structural assignments of the individual residues, NOESY spectra were utilized to establish the sequential connectivity of this primary PAMK backbone. Specifically, strong cross-peaks between A-H1/B-H4 (*δ* 5.02/4.05 ppm), C-H1/A-H4 (*δ* 5.19/4.35 ppm), and D-H1/C-H2 (*δ* 4.93/4.06 ppm) indicated an interconnected backbone sequence consisting of →4)-*α*-D-GalpA-(1→, →4)-*β*-D-Galp-(1→, →2)-*α*-L-Rhap-(1→, and →3,4)-*α*-D-GalpA-(1→. Furthermore, NOESY correlations such as H-H1/G-H6 (*δ* 4.41/3.85, 3.97 ppm) and G-H1/D-H3 (*δ* 4.42/4.07 ppm) elucidated that side chains composed of arabinose and galactose residues are attached to the →3,6)-*β*-D-Galp-(1→ branching units, which as a whole are linked to the O-3 position of the →3,4)-*α*-D-GalpA-(1→ backbone residue. Supported by consistent methylation data, these spatial correlations successfully elucidated the complete linkage arrangement of this predominant acidic heteropolysaccharide fragment.

### Effects of PAMK on cytokine and necroptosis mRNA expression in thymus of goslings after CTX treatment

**Figure 4.**
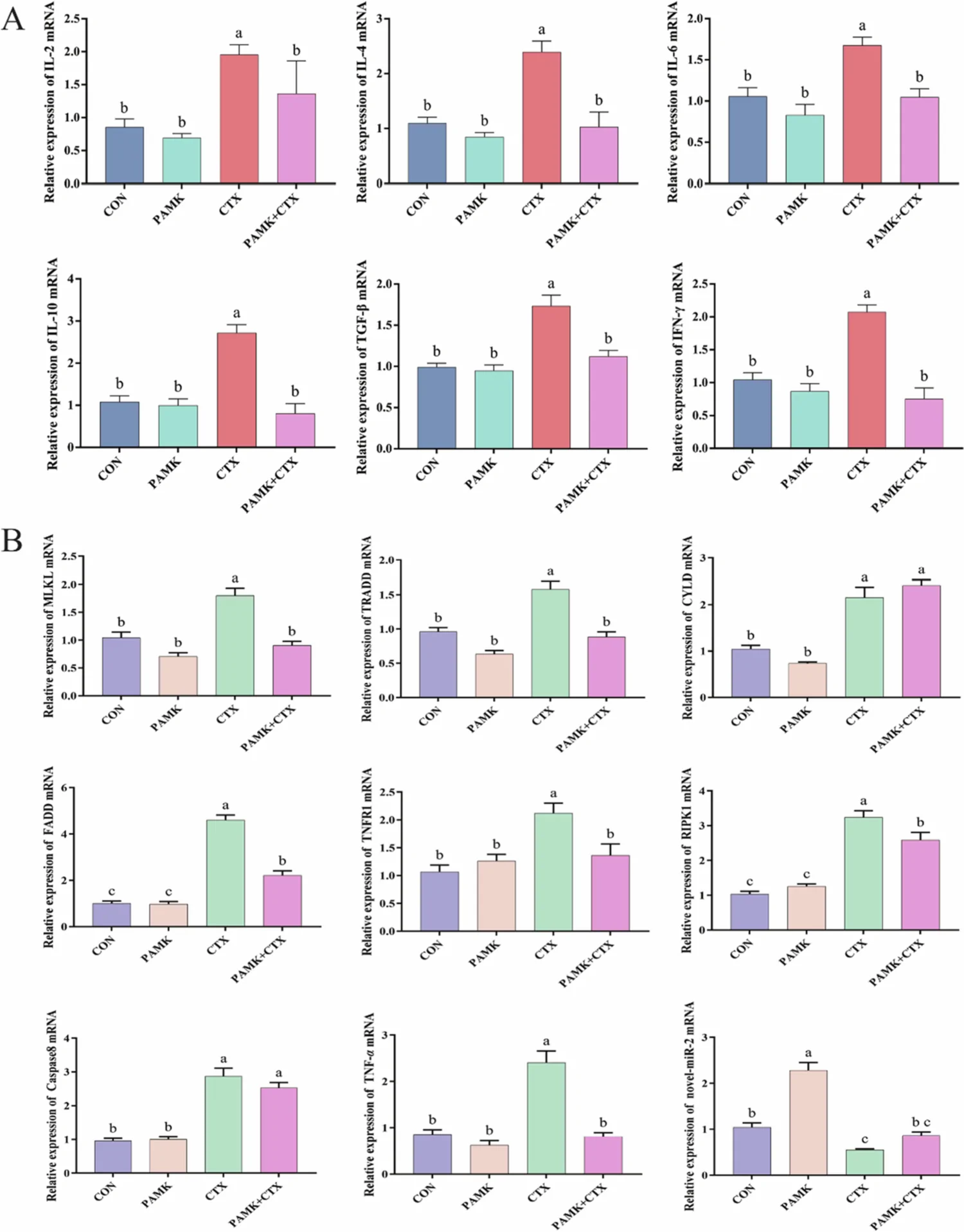
Effects of PAMK on cytokine expression, necroptosis-related genes, and novel-miR-2 in the thymus of goslings following CTX treatment. (A) Relative mRNA expression of cytokines in thymus. (B) Relative mRNA expression of necroptosis-related genes and novel-miR-2 in thymus. Data are presented as mean ± SEM, *n* = 5. Different letters indicate statistically significant differences (*P* < 0.05).

As shown in Figure 4A, compared with the CON group, the CTX group exhibited significantly elevated mRNA expression levels of IL-2, IL-4, IL-6, IL-10, TGF-*β*, and IFN-*γ* (*P* < 0.05). In contrast, PAMK + CTX treatment effectively normalized cytokine transcription: the relative mRNA levels of these cytokines were significantly reduced compared with the CTX group (*P* < 0.05) and were not different from those of the CON group (*P* > 0.05). These findings suggest that PAMK suppresses the CTX-induced transcriptional upregulation of cytokines in gosling thymus tissue.

As shown in Figure 4B, CTX treatment markedly increased the mRNA expression of MLKL, TRADD, CYLD, FADD, TNF-*α*, TNFR1, Caspase8, and RIPK1, while significantly reducing the expression of novel-miR-2 (*P* < 0.05). Compared with the CTX group, PAMK + CTX treatment significantly downregulated the transcription of necroptosis-related genes, with the exception of CYLD and caspase-8 (*P* < 0.05), and simultaneously restored novel-miR-2 expression (*P* > 0.05). Together, these results demonstrate that CTX activates the transcription of necroptosis-related genes in the thymus of goslings, whereas PAMK suppresses this induction. Moreover, PAMK reverses the CTX-mediated downregulation of novel-miR-2, suggesting a potential negative regulatory relationship between novel-miR-2 and necroptosis-associated genes.

### Effects of PAMK on cytokine and necroptosis protein expression in the thymus of goslings following CTX treatment

**Figure 5.**
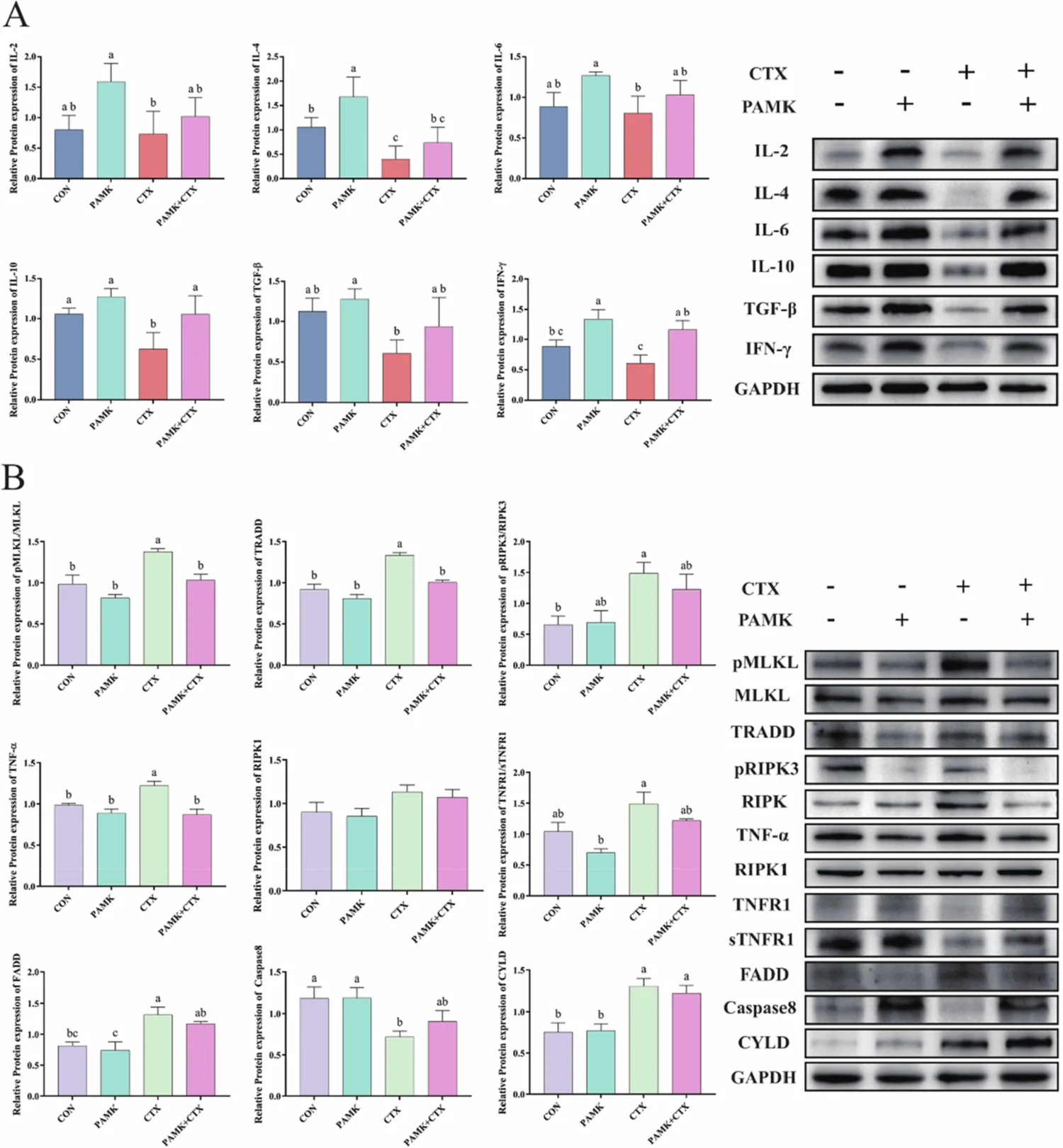
Effects of PAMK on cytokine and necroptosis-related protein expression in the thymus of goslings following CTX treatment. (A) Relative protein expression of cytokines in thymus. (B) Relative protein expression of necroptosis-related pathway proteins in thymus (*n* = 3).

As shown in Figure 5A, compared with the CON group, the CTX group exhibited significantly reduced protein expression of IL-4 and IL-10 (*P* < 0.05). In contrast, the PAMK + CTX group displayed markedly increased protein expression of IL-10 and IFN-*γ* relative to the CTX group (*P* < 0.05). No significant difference was observed in IL-2 protein expression among the groups (*P* > 0.05). However, IL-2 expression in the PAMK group was higher than that in the other three groups. These results suggest that PAMK restores thymic immune function by upregulating cytokine protein expression and counteracting the CTX-induced suppression of cytokine production in gosling thymus.

As shown in Figure 5B, CTX treatment significantly increased the protein expression of TRADD, TNF-*α*, FADD, and CYLD, the ratios of TNFR1 to sTNFR1 and pRIPK3 to RIPK3, and the phosphorylation level of MLKL, while significantly decreasing caspase-8 protein expression (*P* < 0.05). Compared with the CTX group, PAMK + CTX treatment significantly reduced the protein expression of pMLKL/MLKL, TRADD, and TNF-*α* (*P* < 0.05), accompanied by a downward trend in the TNFR1/sTNFR1 ratio and an upward trend in caspase-8 expression, though these changes did not reach statistical significance (*P* > 0.05). These findings indicate that PAMK alleviates CTX-induced necroptosis by downregulating necroptosis-related proteins and inhibiting MLKL phosphorylation.

### Predicted dual targeting relationship of novel-miR-2 with MLKL and TRADD

**Figure 6.**
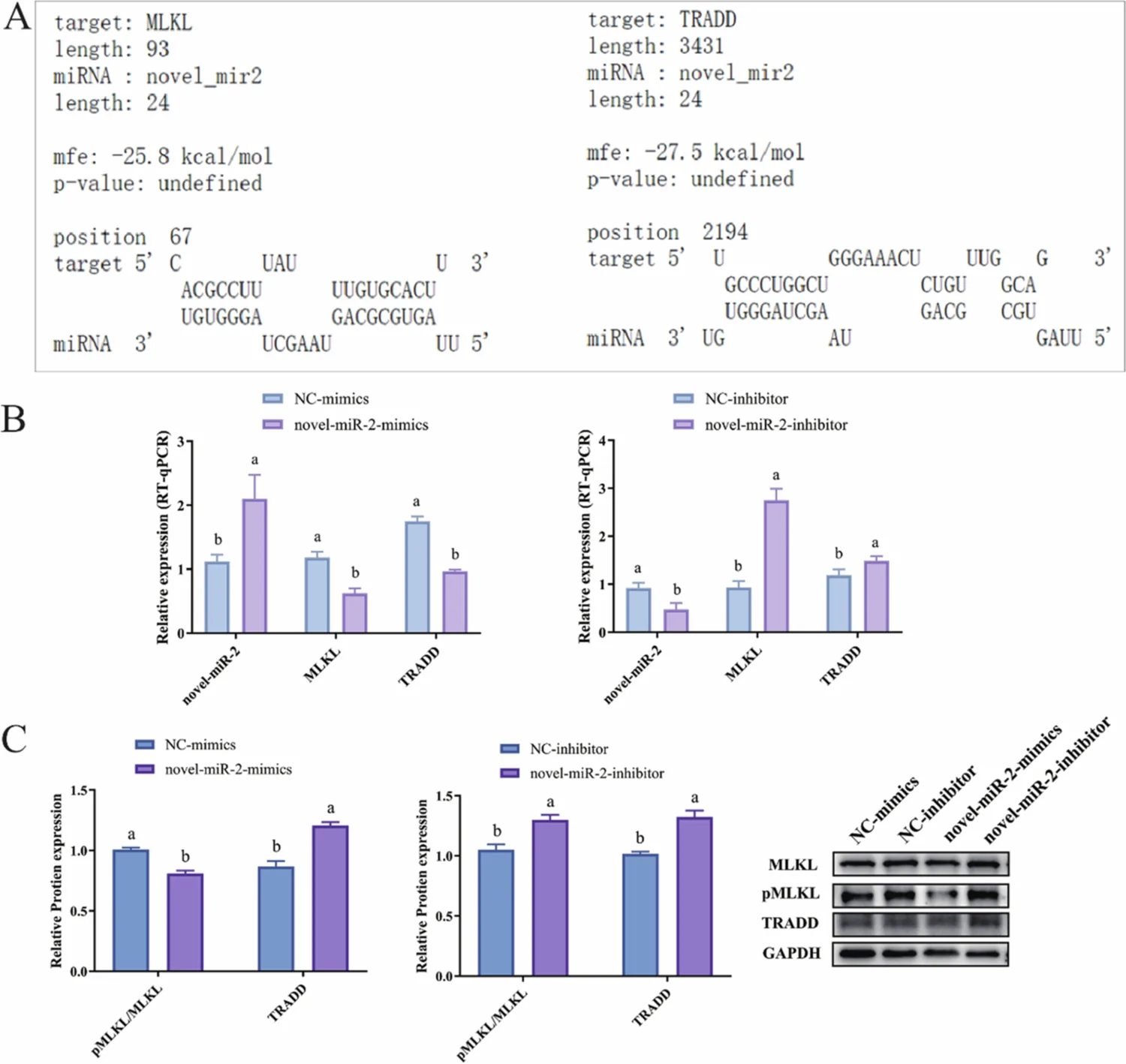
Predicted binding sites of novel-miR-2 with MLKL and TRADD. (A) Effects of novel-miR-2 overexpression or inhibition on the expression levels of the predicted target genes MLKL and TRADD. (B) Comparison of mRNA expression between the novel-miR-2-mimics group and the NC-mimics or NC-inhibitor groups (*n* = 5). (C) Comparison of protein expression between the novel-miR-2-mimics group and the NC-mimics or NC-inhibitor groups (*n* = 3).

As shown in Figure 6A, based on the successfully established animal model of PAMK regulation in CTX-induced thymic necroptosis, we further investigated the underlying mechanism. Referring to our previous work, we selected novel-miR-2 and applied the online prediction tool bibiserv2 to analyze its potential targets and seed region binding sites. The results (Figure 6A) indicated that novel-miR-2 may simultaneously target MLKL and TRADD, two key genes in the necroptosis pathway.

As shown in Figure 6B, compared with the NC-mimics group, the novel-miR-2-mimics group exhibited a significant increase in novel-miR-2 expression (*P* < 0.05), accompanied by a marked reduction in MLKL and TRADD mRNA levels (*P* < 0.05). Conversely, compared with the NC-inhibitor group, the novel-miR-2-inhibitor group displayed significantly increased MLKL and TRADD mRNA expression (*P* < 0.05), while novel-miR-2 expression was significantly decreased (*P* < 0.05).

As shown in Figure 6C, protein expression analysis further confirmed these findings. In the novel-miR-2-inhibitor group, TRADD protein expression and MLKL phosphorylation were significantly elevated compared with the NC-inhibitor group (*P* < 0.05). In contrast, compared with the NC-mimics group, the novel-miR-2-mimics group exhibited a significant reduction in MLKL protein expression but an unexpected increase in TRADD protein expression (*P* < 0.05). Collectively, these results suggest that novel-miR-2 regulates MLKL and TRADD expression. Specifically, the synchronized downregulation of both mRNA and protein levels following novel-miR-2 overexpression suggests that this miRNA primarily modulates its targets by reducing mRNA stability and promoting transcript degradation, rather than solely inhibiting protein translation.

### Protective effects of PAMK against CTX-induced necroptosis and oxidative stress in goose thymocytes

**Figure 7.**
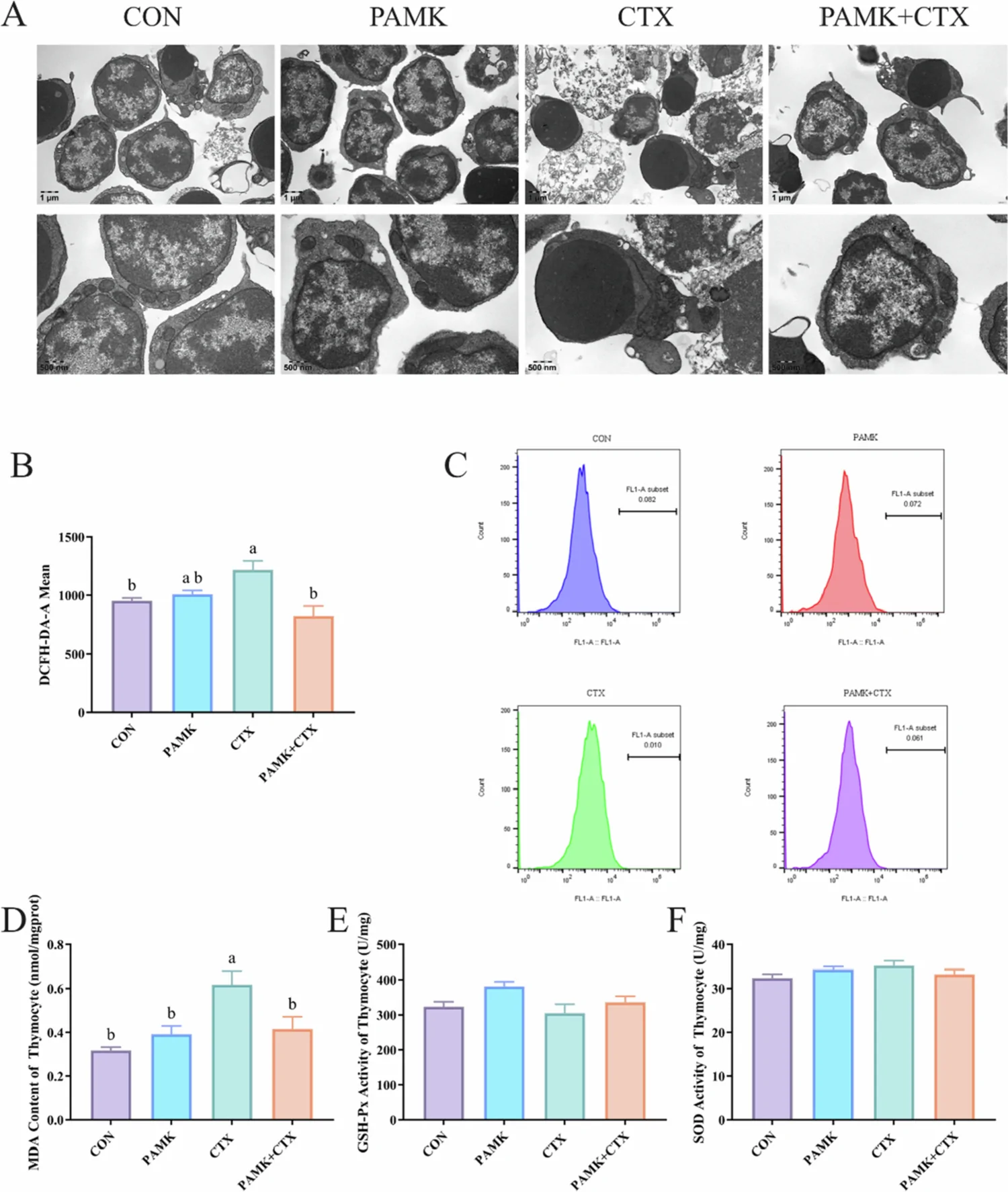
Ultrastructural changes and oxidative stress in goose thymocytes. (A) Transmission electron microscopy of thymocytes (scale bars: 1 μm and 500 nm). (B) ROS levels in thymocytes detected by DCFH-DA staining, with mean fluorescence intensity measured by flow cytometry. (C) Quantification of ROS levels. (D) MDA content (*n* = 5). (E) GSH-Px activity (*n* = 5). (F) SOD activity (*n* = 5).

As shown in Figure 7A, thymocyte in the CON and PAMK groups displayed normal chromatin distribution and morphology, with nearly round cell shapes. In contrast, the CTX group exhibited membrane rupture, organelle leakage, abnormal morphology, chromatin condensation, and features of both necrosis and apoptosis. In addition, remnants of dead cells were observed in low-magnification fields of the CTX group. In the PAMK + CTX group, a small proportion of nuclei appeared shrunken and vacuolated, but overall cell morphology was markedly improved compared with the CTX group. These findings provide in vitro evidence that CTX induces both apoptosis and necrosis in thymocytes, whereas PAMK alleviates cell death and preserves cellular morphology.

As shown in Figure 7B and D, CTX treatment significantly increased ROS levels and MDA content in thymocytes, while slightly decreasing GSH-Px activity (*P* < 0.05). Compared with the CTX group, the PAMK + CTX group exhibited significantly reduced ROS levels and MDA content, accompanied by a slight increase in GSH-Px activity. No significant differences in SOD activity were observed among the four groups (*P* > 0.05). These results indicate that CTX triggers oxidative stress in thymocytes primarily by elevating ROS and MDA rather than by markedly suppressing GSH-Px or SOD activity. PAMK partially mitigates CTX-induced oxidative stress in thymocytes.

### Effect of PAMK on cytokine expression induced by CTX in thymocyte

**Figure 8.**
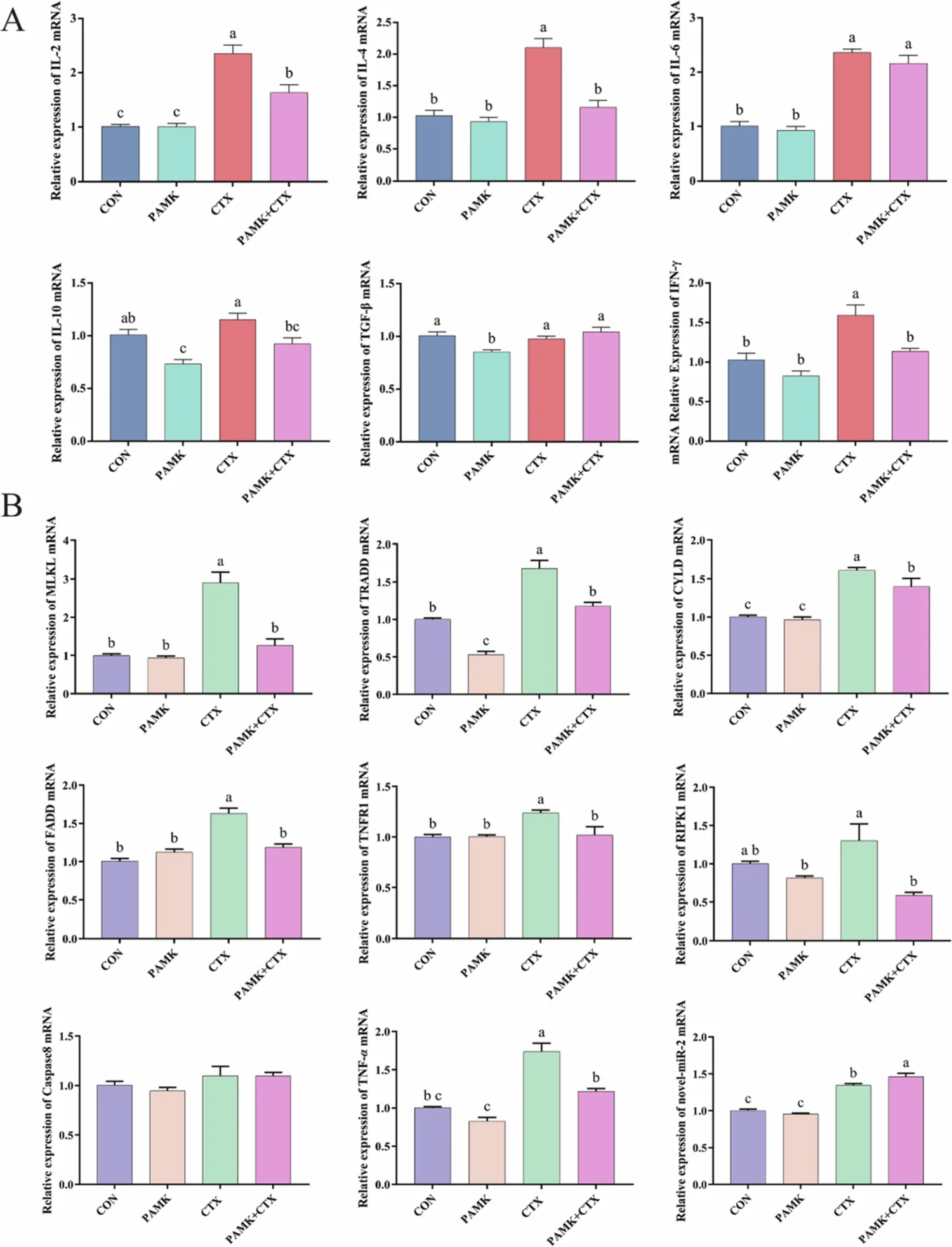
Effect of PAMK on the mRNA expression of cytokines, necroptosis pathway-related genes, and novel-miR-2 induced by CTX in thymocyte. (A) Relative mRNA expression levels of cytokines in thymocyte (*n* = 5). (B) Relative mRNA expression levels of necroptosis pathway-related genes and novel-miR-2 in thymocyte (*n* = 5).

As shown in Figure 8A, compared to the CON group, the mRNA expression levels of IL-2, IL-4, IL-6, and IFN-*γ* were significantly elevated in the CTX group (*P* <  0.05), while IL-10 expression was slightly increased. In contrast, in the PAMK + CTX group, the mRNA expression levels of IL-2, IL-4, IL-10, and IFN-*γ* were significantly reduced compared to the CTX group (*P* < 0.05). These results confirm, in vitro, that CTX promotes the transcription of specific cytokines in thymus tissue, while PAMK regulates cytokine transcription, maintaining their expression at baseline levels.

Figure 8B shows that, compared to the CON group, the mRNA expression levels of MLKL, TRADD, CYLD, FADD, TNF-*α*, TNFR1, caspase-8, and novel-miR-2 were significantly increased in the CTX group (*P* < 0.05). In the PAMK + CTX group, the mRNA expression levels of these genes, along with RIPK1, were significantly decreased compared to the CTX group (*P* < 0.05). Notably, the mRNA expression levels of caspase-8 and novel-miR-2 were significantly elevated (*P* < 0.05). These findings further support the notion that CTX induces the transcription of genes associated with the programmed necrosis pathway in goose thymocyte, while PAMK inhibits the CTX-induced upregulation of these genes.

### The effect of PAMK on the expression of CTX-induced necroptosis-related genes and novel-miR-2 in Thymocyte

**Figure 9.**
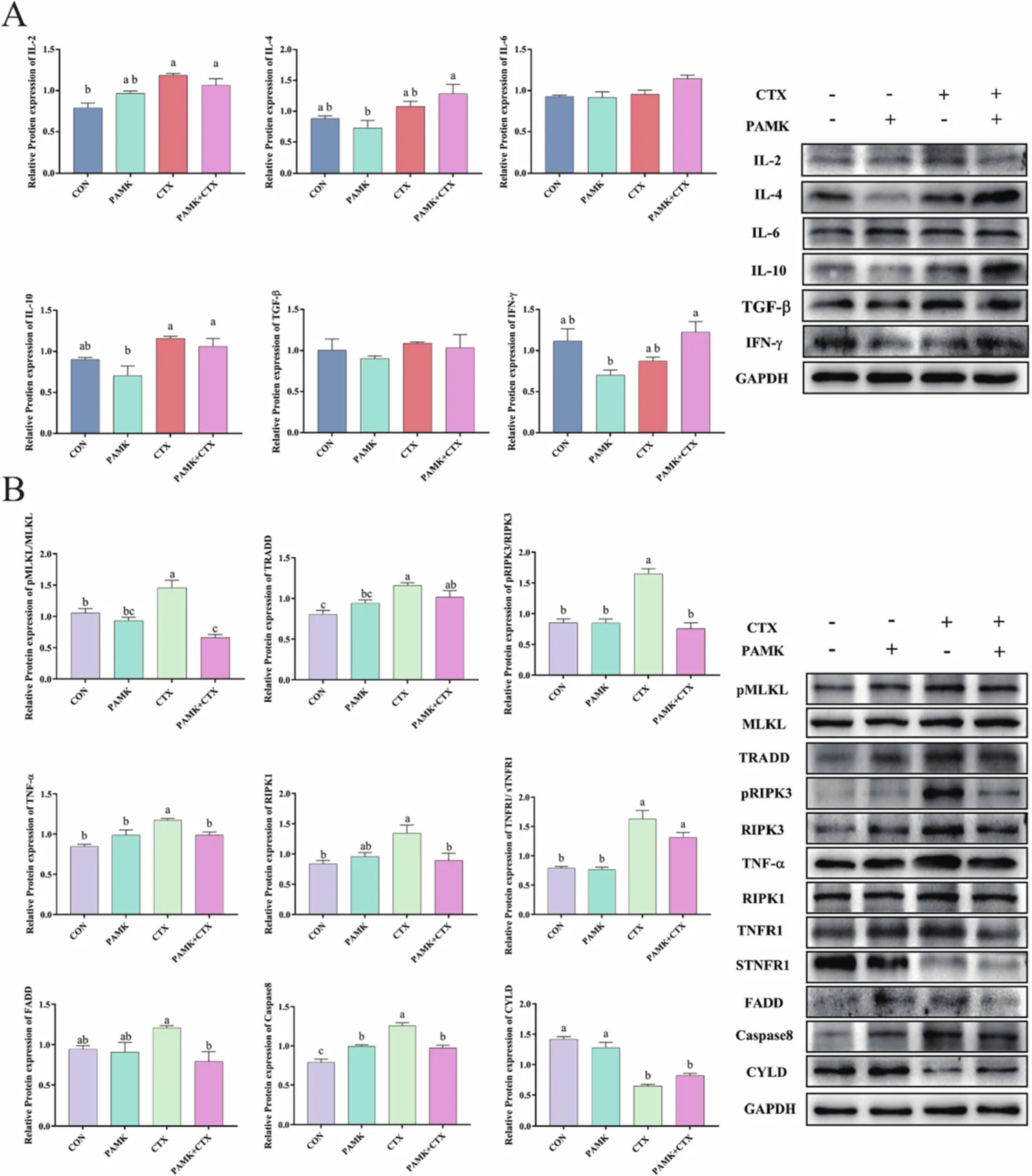
Effect of PAMK on the relative expression of cytokines and proteins related to programmed necrosis pathway in thymocyte induced by CTX. (A) Relative expression of thymic cytokine proteins (*n* = 3); (B) Relative expression of proteins associated with the programmed necrosis pathway in thymocyte (*n* = 3).

As shown in Figure 9A, compared to the CON group, the protein expression level of IL-2 in the CTX group was significantly increased (*P* < 0.05), while the protein expression levels of IL-4 and IL-10 showed a slight increase. Compared to the CTX group, the PAMK + CTX group exhibited a significant increase in the protein expression level of IFN-*γ* (*P* < 0.05), with IL-4 and IL-6 levels showing an upward trend (*P* > 0.05). These results confirm in vitro that PAMK regulates the expression of cytokine proteins, maintaining immune function.

As shown in Figure 9B, compared to the CON group, the protein expression levels of TRADD, TNF-*α*, RIPK1, and Caspase8 in thymic cells of the CTX group, along with the phosphorylation levels of MLKL and RIPK3, and the protein expression ratio of TNFR1 to sTNFR1, were significantly elevated (*P* < 0.05). In contrast, the protein expression level of CYLD was significantly reduced (*P* < 0.05). Although the protein expression level of FADD did not show a significant increase in the CTX group, it exhibited a general upward trend. Compared to the CTX group, the PAMK + CTX group showed significantly reduced expression levels of TNF-*α*, RIPK1, FADD, and caspase-8, along with decreased phosphorylation levels of MLKL and RIPK3 (*P* < 0.05). The protein expression ratio of TNFR1 to sTNFR1 decreased but did not reach statistical significance (*P* > 0.05), and the expression level of CYLD slightly increased without significant difference (*P* > 0.05). These findings further confirm in vitro that PAMK alleviates CTX-induced necroptosis by downregulating the expression of necroptosis-related proteins and the phosphorylation levels of MLKL and RIPK3.

### PAMK alleviates MLKL and TRADD-induced necroptosis and oxidative stress in goose thymocytes

**Figure 10.**
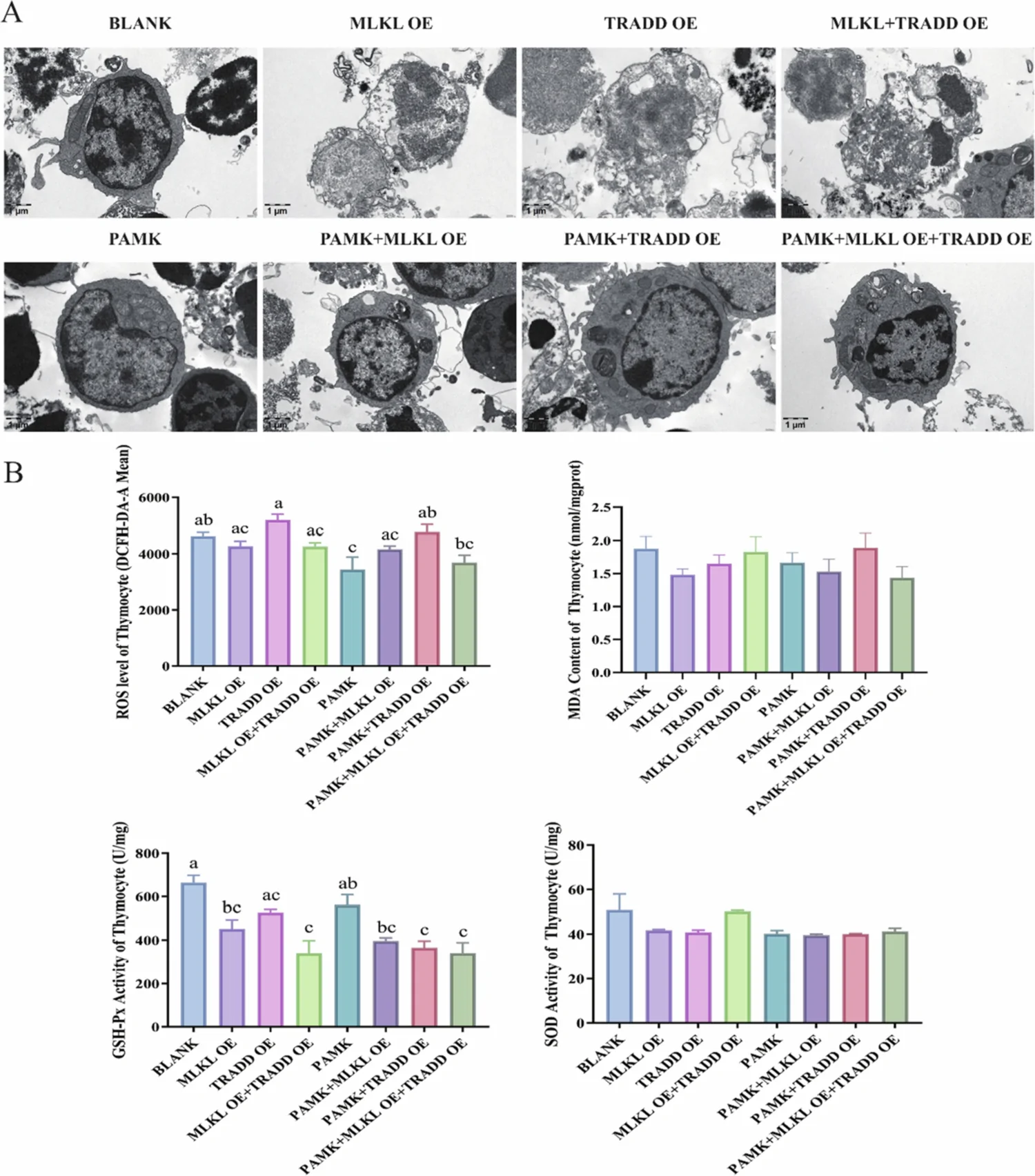
Effects of MLKL and TRADD overexpression on oxidative stress in goose thymocytes. (A) Electron microscopy image of thymocyte (scale bar = 1 μm). (B) Measurement of antioxidant-related indicators in thymocyte (*n* = 5).

The thymocyte were collected for TEM analysis after 48 h of overexpression. As shown in Figure 10A, the chromatin distribution and morphology of the cells in the BLANK and PAMK groups were normal, resembling a round shape. In contrast, cells in the MLKL OE, TRADD OE, and MLKL OE + TRADD OE groups exhibited cell membrane rupture, organelle leakage, morphological abnormalities, chromatin condensation, and features of necrosis. In the PAMK + MLKL OE, PAMK + TRADD OE, and PAMK + MLKL OE + TRADD OE groups, cell membrane rupture and nuclear shrinkage were also observed, accompanied by vacuolation; however, the overall cell morphology was better than in the overexpression groups. These results suggest that overexpression of MLKL and TRADD induces necrosis in goose thymocyte. The in vitro experiments further validated that PAMK alleviates the process of necrosis induced by MLKL and TRADD overexpression, and PAMK restores cell status and improves cell morphology.

As shown in Figure 10B, the ROS levels in the TRADD OE group were increased compared to the BLANK group, whereas ROS levels were significantly reduced in the PAMK group. The GSH-Px activity in the MLKL OE and MLKL OE + TRADD OE groups was significantly decreased (*P* < 0.05). After adding PAMK, ROS levels were reduced to varying degrees in the overexpression groups. These findings indicate that PAMK reduces ROS production and alleviates oxidative stress induced by MLKL and TRADD overexpression in goose thymocyte.

### Impact of PAMK on necroptosis and cytokine gene expression in thymocyte

**Figure 11.**
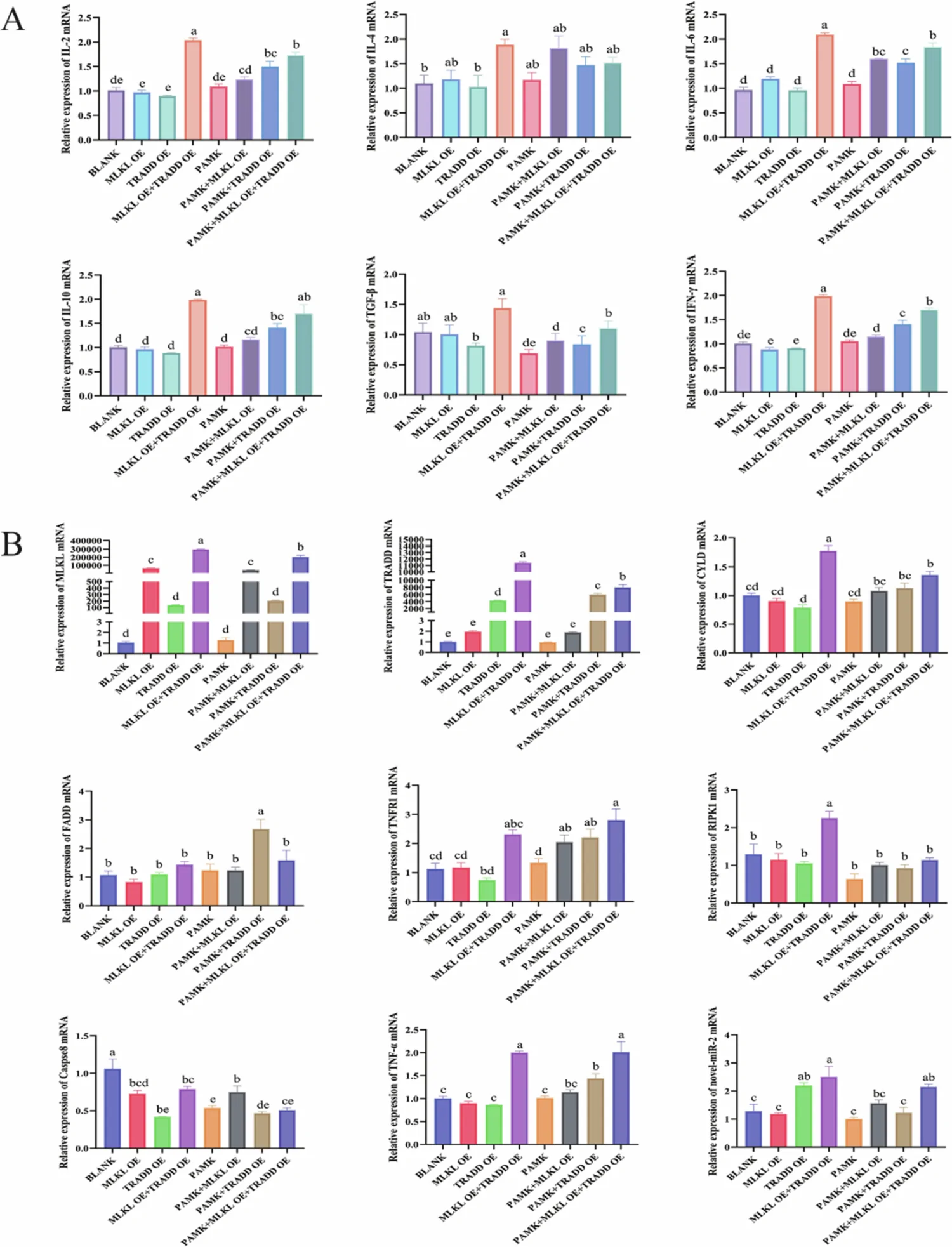
Impact of PAMK on necroptosis and cytokine gene expression in goose thymocyte. (A) Relative mRNA expression levels of cytokines in thymocyte (*n* = 5). (B) Relative mRNA expression levels of necroptosis pathway-related genes and novel-miR-2 in thymocyte (*n* = 5).

As shown in Figure 11A, compared to the BLANK group, the cells in the MLKL OE + TRADD OE group exhibited a significant increase in the mRNA relative expression levels of IL-2, IL-4, IL-6, IL-10, and IFN-*γ* (*P* < 0.05). However, upon the addition of PAMK, the PAMK + MLKL OE + TRADD OE group showed a significant downregulation in the mRNA relative expression levels of IL-2, IL-6, TGF-*β*, and IFN-*γ* (*P* < 0.05). In comparison to the TRADD OE group, the PAMK + TRADD OE group significantly increased the mRNA relative expression levels of IL-2, IL-6, IL-10, TGF-*β*, and IFN-*γ* (*P* < 0.05). Moreover, the PAMK + MLKL OE group was able to modulate the mRNA relative expression levels of IL-2, IL-6, IL-10, TGF-*β*, and IFN-*γ* (*P* < 0.05) when compared to the MLKL OE group.

When MLKL or TRADD was overexpressed individually, their impact on the cytokine mRNA expression levels in goose thymocyte was minimal, with no significant differences observed when compared to the BLANK group (*P* < 0.05). Furthermore, PAMK had a limited inhibitory effect on the elevation of cytokine transcription induced by MLKL and TRADD overexpression.

As shown in Figure 11B, the relative mRNA expression of MLKL significantly increased following MLKL overexpression (*P* < 0.05), and TRADD mRNA levels also significantly increased upon overexpression (*P* < 0.05). When both MLKL and TRADD were overexpressed simultaneously, there was a significant upregulation of the mRNA relative expression of MLKL, TRADD, CYLD, TNF-*α*, RIPK1, and novel-miR-2 (*P* < 0.05). These results indicate successful induction of MLKL and TRADD overexpression in goose thymocyte, along with the transcriptional upregulation of genes associated with necroptosis pathways. Upon adding PAMK, the PAMK + MLKL OE + TRADD OE group showed a significant reduction in the mRNA levels of MLKL, TRADD, CYLD, and RIPK1 compared to the MLKL OE + TRADD OE group (*P* < 0.05). These findings suggest that PAMK effectively alleviates the overexpression of necroptosis-related genes induced by the combined overexpression of MLKL and TRADD in goose thymocyte.

### PAMK regulates cytokine expression and alleviates necroptosis in thymocyte

**Figure 12.**
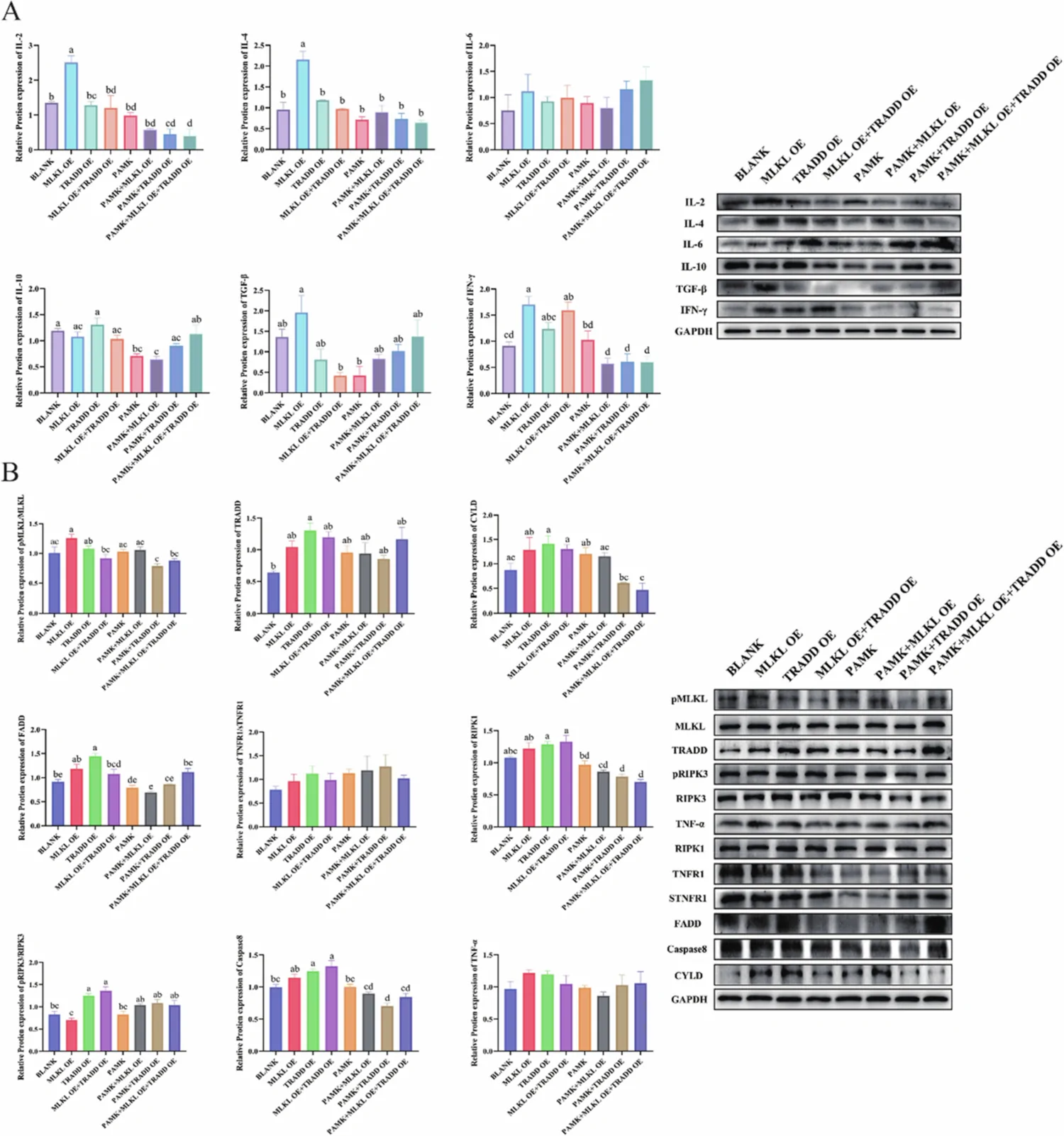
PAMK regulates cytokine expression and alleviates necroptosis in thymocyte. (A) Relative protein expression levels of cytokines in thymocyte (*n* = 3). (B) Relative expression levels of necroptosis pathway-related genes and novel-miR-2 proteins in thymocyte (*n* = 3).

As shown in Figure 12A, compared to the BLANK group, the MLKL OE group exhibited a significant increase in the protein expression levels of IL-2, IL-4, and IFN-*γ* (*P* < 0.05), with a slight increase in IL-6 and TGF-*β* protein expression. In comparison to the MLKL OE group, the PAMK + MLKL OE group showed a significant decrease in the mRNA expression levels of IL-2, IL-4, IL-10, and IFN-*γ* (*P* < 0.05). No significant effects on cytokine expression were observed in the TRADD OE group when compared to the BLANK group. These results suggest that 48 h of MLKL overexpression at the downstream necroptosis pathway leads to an increase in cytokine protein expression in goose thymocyte, and that PAMK has a regulatory effect on the elevated cytokine protein levels induced by MLKL overexpression, thereby alleviating the inflammatory response.

As shown in Figure 12B, compared to the BLANK group, the TRADD OE group exhibited a significant increase in the protein expression levels of TRADD, FADD, and Caspase8, along with a significant increase in the phosphorylation of RIPK3 (*P* < 0.05). Although no significant increase in the phosphorylation of MLKL was observed in the MLKL OE group, a noticeable upward trend was noted. In the simultaneous overexpression of MLKL and TRADD, significant changes were observed in the phosphorylation levels of Caspase8 and RIPK3 (*P* < 0.05), while the expression levels of other necroptosis-related proteins remained unchanged. Following the addition of PAMK, the expression of necroptosis-related proteins was reduced to varying degrees in the transfected cells, and the phosphorylation of MLKL significantly decreased (*P* < 0.05). The experiment demonstrated that PAMK can alleviate necroptosis in goose thymocyte by downregulating the expression of necroptosis-related proteins and reducing the phosphorylation of MLKL.

## Discussion

In this study, we established a CTX-induced immunosuppression model in goslings and systematically evaluated the protective effects of PAMK against thymic necroptosis. For the first time, we comprehensively demonstrated that PAMK alleviates CTX-induced thymic injury through multiple targets and layers, encompassing histopathology, ultrastructure, oxidative stress, biochemical indices, cytokine profiles, miRNA expression, and necroptosis-related proteins (TRADD, RIPK1, RIPK3, MLKL, and phosphorylated MLKL). Collectively, these findings provide mechanistic insights and practical value, supporting the application of PAMK as a green immunoenhancer in poultry production.

We found that necroptosis is a prominent pathological feature of thymic injury, characterized by the loss of cortico-medullary boundaries, mitochondrial swelling, plasma membrane rupture, and amplified inflammation (Xiang et al. 2020). At the molecular level, these hallmarks were further confirmed by the significant upregulation of necroptosis-related markers (RIPK1, RIPK3, MLKL, and *p*-MLKL) and the suppression of caspase-8, indicating activation of the canonical RIPK1/RIPK3/MLKL axis (Bedoui et al. 2020). Crucially, the profound downregulation of Caspase-8 observed in the CTX-treated group provides essential mechanistic insight into the initiation of this process. Caspase-8 is widely recognized as the master molecular switch that dictates the cellular decision between apoptosis and necroptosis. Under normal physiological conditions or typical apoptotic stress, active Caspase-8 directly cleaves and inactivates RIPK1 and RIPK3, thereby dismantling the necroptotic machinery. However, when Caspase-8 activity is compromised or its expression is severely suppressed—as robustly induced by CTX in our model—this regulatory brake is released. The depletion of Caspase-8 creates a highly permissive intracellular environment, allowing RIPK1 and RIPK3 to associate via their RIP homotypic interaction motifs (RHIMs) and assemble into the functional necrosome, ultimately driving MLKL phosphorylation and membrane rupture. By demonstrating that PAMK treatment actively counteracts the CTX-induced depletion of Caspase-8, our data suggest that PAMK not only directly downregulates the necrosome components but also helps maintain the crucial Caspase-8 ‘brake’ to prevent the pathogenic switch toward hyper-inflammatory necroptosis. Similar necroptotic patterns have been reported in thymocytes under pathological stress, including infection and metabolic imbalance, and are increasingly recognized as critical drivers of thymic dysfunction (Yang et al. 2024; Ding et al. 2025). Thus, our model highlights necroptosis as a central mechanism of thymic pathology rather than a simple byproduct of CTX exposure. This finding provides a significant conceptual advance over our previous report on CTX-induced ferroptosis. While ferroptosis primarily mediates intracellular metabolic dysfunction and lipid toxicity, necroptosis is mechanically distinct; it executes cell membrane lysis, thereby serving as the primary engine for massive DAMP release and systemic inflammatory amplification. By establishing that CTX strongly activates necroptosis, we clarify that thymic immune dysfunction is heavily driven by this necroinflammation feed-forward loop.

Most importantly, the current study establishes an unprecedented incremental novelty in two key dimensions compared to our prior work. First, we provide the first comprehensive structural elucidation of the active PAMK fraction (an acidic heteropolysaccharide), explicitly linking its multivalent branched structure to its bioactivity. Second, we uncover a completely distinct post-transcriptional regulatory network. Rather than targeting GPX4 in ferroptosis, we identified that PAMK epigenetically orchestrates the suppression of necroptosis by restoring novel-miR-2 expression. Necroptosis promotes the release of DAMPs and pro-inflammatory cytokines (Abulfadl et al. 2023), fueling a feed-forward loop between cell death and inflammation (Yuan et al. 2019; Ye et al. 2023). Our data therefore robustly support the conclusion that CTX damages the thymus by activating highly inflammatory necroptosis, and that structurally defined PAMK mitigates this specific injury through a novel miRNA-mediated TRADD/MLKL suppression mechanism.

A major finding of our work is that PAMK supplementation effectively attenuated thymic necroptosis. Mechanistically, the TNF/TNFR1-TRADD axis drives mitochondrial ROS accumulation, which stabilizes the necrosome and accelerates downstream membrane rupture (Hanus et al. 2015; Gao et al. 2022). It is important to note that while our data highlight TRADD as a pivotal molecular hub—specifically targeted by novel-miR-2—TRADD typically functions as a scaffold protein to recruit RIPK1 to the activated receptor complex. The classical execution of necroptosis strictly requires the auto- and trans-phosphorylation events driven by RIPK1 kinase activity, which subsequently phosphorylates RIPK3 and MLKL. Although the significant upregulation of total RIPK1 expression and the concurrent elevation in *p*-RIPK3 and *p*-MLKL levels in our CTX model strongly implicate the active engagement of this canonical kinase cascade, TRADD is not universally essential for necroptosis in all biological contexts. Because the current study did not employ specific pharmacological inhibition (e.g. using the RIPK1 kinase inhibitor Necrostatin-1s) or genetic kinase-dead models, we cannot definitively conclude whether RIPK1 kinase activity is strictly indispensable for thymic necroptosis in this specific gosling model. This represents a limitation of our current work and highlights a crucial direction for future investigations to disentangle the scaffolding versus kinase-dependent roles of the necrosome components in avian immunity. Nevertheless, oxidative stress is a critical driver of necroptosis, in which the accumulation of ROS stabilizes the necrosome and accelerates membrane rupture, while MLKL-mediated mitochondrial damage further exacerbates ROS production, thereby reinforcing the vicious cycle between inflammation and necroptosis (Martens et al. 2021; Zhan et al. 2021). PAMK treatment reduced ROS and lipid peroxidation, enhanced antioxidant activity, restored caspase-8 expression, and suppressed *p*-MLKL and TRADD signaling. This dual ‘anti-oxidative-anti-necroptotic’ effect is consistent with previous reports showing that PAMK protects immune organs by alleviating oxidative stress and preventing excessive programmed cell death (Lu et al. 2025). Moreover, PAMK has been widely demonstrated to preserve immune function by stabilizing redox balance, modulating cytokine release, and maintaining T-cell homeostasis under immunosuppressive conditions (Xue et al. 2020). By targeting ROS accumulation and the RIPK1/RIPK3/MLKL signaling pathway, PAMK interrupts the vicious cycle of oxidative stress and necroptosis, thereby preserving thymic architecture and immune competence. These results position PAMK as a promising immunoprotective candidate against necroptosis-driven thymic dysfunction.

Notably, this study characterized the composition of PAMK, revealing that it is an acidic heteropolysaccharide mainly composed of arabinose, galactose, rhamnose, galacturonic acid, and glucose. Compared with previously reported PAMK structures, which are often neutral polysaccharides predominantly rich in glucose or fructose, the PAMK fraction isolated in our study exhibits distinct structural characteristics, particularly its high uronic acid content (~17.7% Gal-UA and Glc-UA) and a highly branched backbone. Such a typical heteropolysaccharide structure has been widely associated with immunomodulatory and antioxidant activities in polysaccharides of plant origin (Li et al. 2022). Previous studies have demonstrated that the extraction method, geographical origin, and harvest season can lead to structural diversity in PAMK, which in turn dictates its pharmacological efficacy (Luo et al. 2022). By clearly defining the chemical composition and backbone configuration of PAMK, we were able to systematically link its structural features with its biological activity. Numerous studies have shown that plant polysaccharides effectively enhance immune function, including intestinal and systemic immunity, while offering the advantages of safety and low toxicity (Sun et al. 2015; Ji et al. 2020; Zhao et al. 2023). Crucially, the specific structural features of our PAMK fraction provide a strong biochemical rationale for its observed biological activities. First, the presence of *α*-D-galacturonic acid (*α*-D-GalpA) confers a polyanionic character to the polysaccharide. Uronic acid-rich acidic polysaccharides are widely recognized to exhibit superior antioxidant and immunomodulatory properties compared to their neutral counterparts (Ji et al. 2020), as the negative charges facilitate stronger electrostatic interactions with pattern recognition receptors on immune cells. Second, our NMR and methylation analyzes identified a complex branching architecture, where side chains are primarily attached to the O-3 position of the GalpA backbone units, alongside further branching at the O-3 and O-6 positions of Galp units. High degrees of branching and the presence of *β*-glycosidic bonds often promote a more extended and flexible spatial conformation in aqueous environments, providing multivalent binding sites that enhance receptor cross-linking and subsequent downstream signaling. This structural flexibility and active site exposure are highly favorable for quenching ROS and mitigating oxidative stress.

Consistently, PAMK has been reported to regulate immune responses, exert antioxidant and anti-aging effects (Yang et al. 2021; Bo et al. 2022; Yu et al. 2023). In addition, the regulation of cytokine networks observed here further supports its immunoprotective role. We found that PAMK modulated cytokines, which are known to maintain immune balance and exert anti-inflammatory functions, consistent with previous findings (Xu et al. 2017; Kai et al. 2022). And we propose that the potent protective effects of PAMK against CTX-induced thymic necroptosis are intrinsically linked to its acidic heteropolysaccharide nature. The uronic acid moieties and the specific branched backbone structurally enable PAMK to scavenge excessive ROS, restore redox balance, and interact with immune sensors to upregulate novel-miR-2, thereby suppressing the TRADD/MLKL necroptotic axis. Thus, the defined structural features of PAMK not only provide a chemical basis for its immunoprotective activity in goslings but also underlie its antioxidant and anti-necroptotic potential.

Growing evidence indicates that miRNAs directly or indirectly regulate necroptosis-related molecules by targeting death pathway components or modulating inflammatory and metabolic cascades, thereby serving as molecular hubs that connect cell death with tissue homeostasis (Cui et al. 2019; Hu et al. 2019; Kai et al. 2022). For example, miR-425-5p has been reported to attenuate sepsis-associated liver injury by negatively regulating RIP1-mediated necroptotic signaling and inflammation (Gu et al. 2020). In this study, we found that novel-miR-2 expression was reduced in the CTX group but restored in the PAMK group. In primary thymocytes, upregulation of novel-miR-2 generally suppressed MLKL transcription and/or pMLKL levels, whereas inhibition of novel-miR-2 had the opposite effect, suggesting that it may act as an endogenous mediator of PAMK's protective function. Combined with target prediction and expression correlation analyzes, our data support the hypothesis that novel-miR-2 negatively regulates the TRADD/MLKL axis, thereby suppressing necroptosis and improving the inflammatory microenvironment. Regarding the regulatory mode of novel-miR-2, our findings indicate that it acts predominantly at the level of mRNA stability. In the canonical miRNA pathway, miRNAs can silence gene expression through either site-specific cleavage of the mRNA or by inhibiting translation efficiency. In this study, the qPCR and Western blot data showed a consistent decrease in both the mRNA transcripts and protein products of TRADD and MLKL upon novel-miR-2 mimics transfection. This dual reduction is a hallmark of mRNA destabilization, suggesting that novel-miR-2 may trigger the recruitment of the deadenylation complex to the 3' UTR of TRADD and MLKL, subsequently leading to the rapid degradation of these target mRNAs. This mode of action ensures a robust and sustained suppression of the necroptotic signaling cascade in gosling thymocytes.

In summary, PAMK, with its heteropolysaccharide structure composed of glucose, arabinose, galactose, and rhamnose, exhibits significant antioxidant and immunomodulatory potential. We demonstrate that CTX exacerbates systemic and local TNF/TRADD-RIPK1 signaling in parallel with oxidative stress, ultimately inducing thymic necroptosis and immune imbalance. By contrast, PAMK mitigates thymic injury through multiple coordinated mechanisms, including antioxidant defense, restoration of receptor balance, modulation of cytokine profiles, and upregulation of novel-miR-2, thereby suppressing necroptosis and restoring thymic homeostasis. These findings not only expand our understanding of CTX-induced immunotoxicity but also provide mechanistic support for the application of PAMK in avian immune health management, while highlighting novel-miR-2 as a potential therapeutic target or biomarker.

## Conclusion

PAMK was identified as an acidic heteropolysaccharide mainly composed of arabinose, galactose, rhamnose, galacturonic acid, and glucose. It possesses a distinctive primary backbone composed of →4)-*α*-D-GalpA-(1→, →4)-*β*-D-Galp-(1→, →2)-*α*-L-Rhap-(1→, and →3,4)-*α*-D-GalpA-(1→, interlinked with highly branched side chains. The unique structural characteristics of PAMK were closely associated with its bioactivity. Functionally, PAMK supplementation effectively suppressed CTX-induced thymic necroptosis by enhancing novel-miR-2 expression, downregulating TRADD and MLKL, reducing oxidative stress, and restoring thymic immune balance. These findings not only provide novel mechanistic insights into thymic necroptosis in poultry but also establish PAMK as a promising plant-derived immunoenhancer with potential applications in sustainable poultry health management.

## Supplementary Material

Figure S4 .docx Figure S4 .docx

supplemental_data (2).docx supplemental_data (2).docx

Figure S1.docx Figure S1.docx

Figure S2 .docx Figure S2 .docx

Figure S3.docx Figure S3.docx

## Supplemental data

The data involved in this study have been uploaded to Figshare https://doi.org/10.6084/m9.figshare.30148594, available for reading and reference by readers.
