# Supplementary material for: Structural characterization of PAMK and its protective effect against thymic necroptosis in goslings
Source: Vet Q. 2026 Sep 1;46(1):2695104. doi: 10.1080/01652176.2026.2695104 (PMC13537054; doi:10.1080/01652176.2026.2695104)
Supplement: Supplementary file 2 [file TVEQ_A_2695104_SM7508.docx]

**Structural characterization of *PAMK* and its protective effect against thymic necroptosis in goslings**

Wanyan Li^13*^, Baili Lu^1*^, Xiangying Zhou^1^, Shirou Pan^1^, Junhao Wei ^2^, Bingxin Li^13^, Nan Cao^13^, Yunmao Huang^13^, Wenjun Liu^13^, Xinliang Fu^13^, Yunbo Tian^13^, Danning Xu^13^

^1^College of Animal Science & Technology, Zhongkai University of Agriculture and Engineering, Guangzhou 510225, China

^2^Faculty of Applied Sciences, Macao Polytechnic University, Macao 999078, China

^3^Guangdong Engineering Technology Research Center of Biosafety and Intelligent Control for Aquatic Animals Diseases

^*^These authors contributed equally to this work.

**Table S1 Thymocytes CTX modeling and grouping**

| Group | PAMK（μg/mL） | CTX（μM） |
| --- | --- | --- |
| CON | 0 | 0 |
| PAMK | 25 | 0 |
| CTX | 0 | 50 |
| PAMK+ CTX | 25 | 50 |

**Table S2 novel-miR-2 oligonucleotides sequences**

| Gene | Sequence (5' - 3') / Product Number |
| --- | --- |
| novel-miR-2 mimics | F：uuagugcgcaguaagcuagggugu |
|  | R：aaTcacgcgtcattcgatcccaca |
| novel-miR-2 inhibitor | aaTcacgcgtcattcgatcccaca |
| NC-mimics | miR1N0000001-1-5 |
| NC-inhibitor | miR2N0000001-1-5 |

**Table S3 MLKL and TRADD Overexpression Grouping and Treatment**

| Group | PAMK(μg/mL) | MLKL(ng/mL) | TRADD(ng/mL) |
| --- | --- | --- | --- |
| BLANK | 0 | 0 | 0 |
| MLKL OE | 0 | 1000 | 0 |
| TRADD OE | 0 | 0 | 1000 |
| MLKL OE+TRADD OE | 0 | 1000 | 1000 |
| PAMK | 25 | 0 | 0 |
| PAMK+MLKL OE | 25 | 1000 | 0 |
| PAMK+TRADD OE | 25 | 0 | 1000 |
| PAMK+MLKL OE+TRADD OE | 25 | 1000 | 1000 |

**Table S4 Primer sequences**

| Gene | Sequences（5´ - 3´） | Accession Number |
| --- | --- | --- |
| *IL-2* | F: CCAGGAACGGGATGCAATATCTGTG | XM_048078148.1 |
|  | R: CTCAGGAAGTTGGTCAGCTCTTGG |  |
| *IL-4* | F: GGCATCTACCTCAACTTGCT | XM_013175717.2 |
|  | R: CTCTTTCGCTACTCGTTGGA |  |
| *IL-6* | F: ACGATAAGGCAGATGGTGAT | XM_048070285.1 |
|  | R: TCCAGGTCTTATCCGACTTC |  |
| *IL-10* | F: ATCATGACATGGACCCGGTA | XM_048071022.1 |
|  | R: ATTGCTCCATGACAGTTGCT |  |
| *IFN-γ* | F: CCAGATTGTTTCCCTGTACTTG | XM_013198313.2 |
|  | R: CATCAGAAAGGGTGTCTCTCA |  |
| *TGF-β* | F: CATCACAGAGACAGGAACCTT | XM_052685384.1 |
|  | R: CTTTCACATCACCACTGGAA |  |
| *MLKL* | F: CCGTGTGCTTGCAAAACGTA | XM_048078694.1 |
|  | R: GCAAGGAGCATCTCCCTCTC |  |
| *TRADD* | F: CCCTGCGAGATCCTGTCATC | XM_048078680.1 |
|  | R: TTTTCTTCCAGGGCTGCGA |  |
| *FADD* | F: TCAACTTCCTCCTCGAGCAG | XM_013194898.2 |
|  | R: GAAAAGCCAAGTTTCCGCA |  |
| *CYLD* | F: TGAAGACACTCCCAGGCAAT | XM_048075364.1 |
|  | R: TGGCAGGTCTTTAGGGAGTG |  |
| *Caspase8* | F: GATGCTCGCCAGAAAGGT | XM_013187587.2 |
|  | R: AGGTAGCCATGCCCAAGA |  |
| *RIPK1* | F: GGTGAGAGAGAACTTGGCCA | NM_001310372.1 |
|  | R: CCTTCCCTCATTCCCCACTT |  |
| *TNFR1* | F: AAAAGACTGTGAACGGCCTG | XM_048047600.1 |
|  | R: GGGTCTCTACTATCTGCTGG |  |
| *TNF-α* | F: GTTCTATGACCGCCCAGTTC | XM_046927265.1 |
|  | R: CACACGACAGCCAAGTCAA |  |
| *β-actin* | F: GCACCCAGCACGATGAAAAT | XM_013174886.1 |
|  | R: GACAATGGAGGGTCCGGATT |  |
| *novel-miR-2* | F: ACACTCCAGCTGGGTTAGTGCGCAGTAAGCTAG |  |
|  | R: CTCAACTGGTGTCGTGGA | / |
|  | RT: CTCAACTGGTGTCGTGGAGTCGGCAATTCAGTTGAGACACCCTA | |
| *U6* | F: CTCGCTTCGGCAGCACA | / |
|  | R: AACGCTTCACGAATTTGCGT |  |
|  | RT: AACGCTTCACGAATTTGCGT |  |

**Table S5 Chemical shifts of ^1^H and ^13^C for the sugar residues.**

| Code | Glycosyl residues | Chemical shifts(ppm) | | | | | |
| --- | --- | --- | --- | --- | --- | --- | --- |
|  |  | H1/C1 | H2/C2 | H3/C3 | H4/C4 | H5/C5 | H6/C6 |
| H | →3,6)-β-D-Gal*p*-(1→ | 4.42 | 3.48 | 3.65 | 4.04 | 3.83 | 3.97,3.85 |
|  |  | 103.31 | 70.84 | 80.38 | 70.18 | 73.59 | 69.40 |
| I | →4)-α-D-Glc*p*-(1→ | 5.32 | 3.52 | 3.87 | 3.59 | 3.71 | 3.75,3.66 |
|  |  | 100.09 | 71.72 | 73.44 | 76.8 | 72.82 | 61.15 |
| J | →3)-α-L-Rha*p*-(1→ | n.d | 4.14 | 3.81 | 3.31 | 3.78 | 1.25 |
|  |  | n.d | 70.38 | 81.37 | 72.82 | 69.49 | 16.83 |
| K | →4)-α-D-Gal*p*A | 5.22 | 3.76 | 3.9 | n.d | n.d | / |
|  |  | 92.22 | 68.21 | 68.82 | n.d | n.d | n.d |
| L | →4)-β-D-Gal*p*A | 4.49 | 3.42 | 3.62 | n.d | n.d | / |
|  |  | 96.09 | 71.53 | 68.24 | n.d | n.d | n.d |
