# Supplementary material for: Structural characterization of PAMK and its protective effect against thymic necroptosis in goslings
Source: Vet Q. 2026 Sep 1;46(1):2695104. doi: 10.1080/01652176.2026.2695104 (PMC13537054; doi:10.1080/01652176.2026.2695104)
Supplement: Supplementary file 3 [file TVEQ_A_2695104_SM7509.docx]

**Figure S1 Purification of PAMK and Monosaccharide Composition**. The molecular weight distribution curve of the purified PAMK fraction determined by HPGPC.
